# The Early History of Heliospheric Science and the Spacecraft That Made It Possible

**DOI:** 10.1007/s11214-022-00900-8

**Published:** 2022-05-25

**Authors:** G. P. Zank, V. Sterken, J. Giacalone, E. Möbius, R. von Steiger, E. S. Stone, S. M. Krimigis, J. D. Richardson, J. Linsky, V. Izmodenov, B. Heber

**Affiliations:** 1 Center for Space Plasma and Aeronomic Research (CSPAR), University of Alabama in Huntsville, Huntsville, AL 35805 USA; 2 Department of Space Science, University of Alabama in Huntsville, Huntsville, AL 35805 USA; 3 ETH Zürich, Zürich, Switzerland; 4 Department of Planetary Science, University of Arizona, Tucson, USA; 5 University of New Hampshire, Durham, USA; 6 International Space Science Institute, Bern, Switzerland; 7 California Institute of Technology, Pasadena, USA; 8 Applied Physics Laboratory, Johns Hopkins University, Baltimore, USA; 9 Kavli Center for Astrophysics and Space Science, Massachusetts Institute of Technology, Cambridge, USA; 10 JILA, University of Colorado and NIST, Boulder, CO 80309 USA; 11 Center for Fundamental and Applied Mathematics, Lomonosov Moscow State University, Moscow, Russia; 12 Space Research Institute (IKI) Russian Academy of Sciences, Moscow, Russia; 13 University of Kiel, Kiel, Germany

**Keywords:** Heliosphere, Interstellar medium

## Abstract

Our understanding of the interaction of the large-scale heliosphere with the local interstellar medium (LISM) has undergone a profound change since the very earliest analyses of the problem. In part, the revisions have been a consequence of ever-improving and widening observational results, especially those that identified the entrance of interstellar material and gas into the heliosphere. Accompanying these observations was the identification of the basic underlying physics of how neutral interstellar gas and interstellar charged particles of different energies, up to and including interstellar dust grains, interacted with the temporal flows and electromagnetic fields of the heliosphere. The incorporation of these various basic effects into global models of the interaction, whether focused on neutral interstellar gas and pickup ions, energetic particles such as anomalous and galactic cosmic rays, or magnetic fields and large-scale flows, has profoundly changed our view of how the heliosphere and LISM interact. This article presents a brief history of the conceptual and observation evolution of our understanding of the interaction of the heliosphere with the local interstellar medium, up until approximately 1996.

## Introduction

At a press conference held in Washington D.C. on 12 September 2013, the Principal Investigator of the Voyager Interstellar Mission, Dr E.C. Stone, announced that the Voyager 1 (V1) spacecraft had crossed the heliopause a year earlier on 25 August 2012, entering the Very Local Interstellar Medium (VLISM). It is now generally but not universally accepted that V1 is in interstellar space (Stone et al. 2013; Krimigis et al. 2013; Burlaga et al. 2013; Gurnett et al. 2013), offering an unprecedented opportunity to study in situ basic plasma physical processes governing the interstellar medium (ISM). That the Voyager 1 spacecraft now finds itself in interstellar space is an event of enormous historical import as humankind exits its solar neighborhood, beginning another epoch of extraordinary discovery science. Six years and two months later, on 5 November 2018, the twin Voyager 2 (V2) spacecraft observed a sharp decrease in the intensity of low-energy ions and a simultaneous increase in the intensity of galactic cosmic rays. This signature moment, much like the Voyager 1 crossing, indicated that V2 too had exited the heliosphere, crossing the heliopause to enter interstellar space. Humankind is now exploring *in situ* our local region of the galaxy with a pair of widely separated spacecraft that are returning extraordinary, possibly once-in-a-lifetime, discoveries that were never anticipated with the launch of both in 1977. This article reviews briefly the history of the science and the spacecraft missions that led up to these historic moments. We restrict our attention to the time prior to and including $\sim 1996$ when the first of the modern models of the large-scale heliosphere were beginning to be developed and when the first of the major heliospheric boundaries, the so-called hydrogen wall or H-wall, was discovered (although we interpret 1996 with some liberalness). The year 1995 was the birth of the International Space Science Institute (ISSI) and the first of its workshops, which was dedicated to the physics of the large-scale heliosphere, an event in which many of the coauthors of this historical review participated.

Shortly after the first models of the solar wind were presented (Parker 1958), it was recognized that the expanding solar wind should carve a bubble in the surrounding interstellar medium. The “bubble” has since come to be termed the heliosphere. These early models, (Davis 1955; Parker 1961, 1963; Axford et al. 1963; Baranov et al. 1971), explored relatively simple gas dynamic 2D models interacting with a gas dynamic description of the local ISM (LISM). Progress beyond these models was relatively slow, in large part because of the paucity of suitable observations (Axford 1972). In looking over the field from the perspective of today, there have been several quite clearly identifiable major observations and/or theoretical advances that propelled the field forward substantially. The first of these that changed the status of the field considerably was the discovery by OGO 5 (Thomas and Krassa 1971; Bertaux and Blamont 1971) that interstellar gas can penetrate the inner solar system. The existence of such a wind of interstellar neutral atoms through the heliosphere, experiencing gravitational focussing, had already been predicted by Fahr (1968). The possibility that the interstellar gas is comprised of a substantial neutral component led to extensive studies investigating the entrance of neutral interstellar gas into the solar wind. It was soon recognized that neutral interstellar hydrogen is the dominant (by mass) constituent of the solar wind beyond an ionization cavity of $\sim 6\text{--}10$ astronomical units (AU) in the upwind direction (the direction antiparallel to the incident interstellar wind) (e.g., Adams and Frisch 1977). Subsequently, UV backscatter observations led to the discovery of interstellar neutral He in the heliosphere (Weller and Meier 1974). The second important realization was that the neutral hydrogen is coupled weakly to the solar wind plasma via resonant charge exchange – a coupling that leads to the production of pickup ions (PUIs) that eventually dominate the internal energy of the distant solar wind. This led to extensive studies of the effects of PUIs on the large-scale solar wind flow, primarily in the context of 1D models of the extended solar wind, and well summarized by Holzer (1979, 1989). The third important advance was the recognition that the resonant charge exchange coupling of plasma and neutral H each influences the other (Wallis 1975, 1984) with the implication that the boundaries separating the heliosphere from the LISM might effectively filter the entrance of neutral H into the heliosphere (Baranov et al. 1979; Ripken and Fahr 1983; Fahr and Ripken 1984). In addition, as had been established in the 1D models showing the effect of PUIs on the extended solar wind, the entrance of neutral H into the heliosphere effectively reduced the ram pressure of the solar wind and hence modified the location of the various boundaries. Fourth was the observational *in situ* discovery and measurement of interstellar PUIs, representing the first direct detection of gas of interstellar origin in the heliosphere. Somewhat serendipitously, Moebius et al. (1985) made the first measurement of pickup ions of interstellar origin when interstellar $\text{He}^{+}$ was discovered with the SULEICA instrument on AMPTE IRM, a consequence of the first AMPTE Lithium ion release into the solar wind (Möbius et al. 1986). Subsequently, the SWICS instrument on the Ulysses spacecraft (Gloeckler et al. 1993, 1994b) discovered $\text{H}^{+}$, $\text{O}^{+}$, $\text{Ne}^{+}$ pickup ions, and shortly thereafter inner source pickup ions. In addition, Ulysses provided a much improved understanding of the 3D structure of the heliosphere (Phillips et al. 1995). Fifth was the first direct observations of interstellar neutral atoms made by Witte et al. (1993) using the Ulysses GAS experiment, and extensively reviewed by Witte et al. (1996). A sixth important result was the discovery by Pioneer 10, the IMPS 5 and 7, and subsequently by the Voyager 1 and 2 spacecraft, of the anomalous cosmic ray (ACRs) component (Garcia-Munoz et al. 1973; Hovestadt et al. 1973; McDonald et al. 1974; Christian et al. 1988) and the subsequent recognition that PUIs are the source of ACRs (Fisk et al. 1974), although the precise mechanism whereby some PUIs increase their energy by 4–6 orders of magnitude is not yet settled. Finally, the seventh major set of results was the separate prediction of the hydrogen wall (H-wall) using different approaches by Baranov et al. (1991), Baranov and Malama (1993) and Pauls et al. (1995), Zank et al. (1996d) and the serendipitous discovery using Lyman-$\alpha $ absorption measurements by Linsky and Wood (1996) and verified by Gayley et al. (1997). This was the discovery of the first of the boundaries separating the heliosphere from the very local interstellar medium.

A resurgence in the field occurred in the mid-1990s, driven in part by the increasing expectation that the now renamed Voyager Interstellar Mission spacecraft would soon cross the first of the heliospheric boundaries, viz., the heliospheric termination shock (HTS). Much of the impetus behind the new activity came from the development of new and sophisticated models that incorporated the coupling of plasma and neutral H, a cosmic ray gradient that continued to increase with heliocentric distance, and very curious observations, beginning in 1984 (Kurth et al. 1984), of mysterious radio emissions that appeared to originate from very large distances ahead of the then current Voyager 1 and 2 locations. This marked the beginning of the modern era of exploration of the interaction of the solar wind with the LISM, marked by increasingly sophisticated models and theory together with a plethora of observations made by the Voyager 1 and 2 spacecraft as they approached and crossed the boundaries of the heliosphere and then by increasingly sophisticated remote observations, beginning with Lyman-$\alpha$ observations made by the STIS instrument on board the Hubble Space Telescope (Linsky and Wood 1996), followed by the observation of Energetic Neutral Atoms (ENAs) created in the distant heliosphere and very local ISM (VLISM) by the Interstellar Boundary Explorer (IBEX) mission (McComas et al. 2009) and by the Cassini mission (Krimigis et al. 2009). We address the beginnings of these developments, but not those that occurred after about 1996, and focus instead on the prior history of the field. Extensive reviews of the early history of the solar wind interaction with the LISM can be found in Axford (1972) and Zank (1999a). Reviews reflecting more recent developments are those by Zank (2015) and Izmodenov et al. (2009).

The paper is organized according to a general discussion of the basic science up until about 1996 (Sect. 2, Basic Science), followed by a brief history of the primary spacecraft that made possible the observations upon which we rely today (Sect. 3, The Spacecraft), and we conclude with Sect. 4, The ISSI Contribution.

## Basic Science: A History Until $\sim 1996$

### Interaction of Interstellar Hydrogen and Helium with the Solar Wind

Interstellar neutral gas flows into the heliosphere relatively unimpeded and can penetrate to within several AU of the Sun. Neutral atoms scatter solar radiation resonantly so that the distribution of Hydrogen (H) and Helium (He) in the heliosphere can be studied by observing sky background radiation in HI $\lambda 1216$ and HeI $\lambda 584$. The basic interactions of neutral H and He with the solar wind plasma are tabulated in Table 1 of Zank (1999a), these being resonant charge exchange between neutral H and protons, photoionization, H-$\text{H}^{+}$, H-H, e-H, and H-$\text{H}^{+}$ Coulomb collisions, electron impact ionization, and recombination. Compared to elastic H-H and H-p collisions, the charge-exchange reaction H-$\text{H}^{+}$ dominates, as discussed by Izmodenov et al. (2000).

The distribution of LISM neutral hydrogen or Helium drifting through the heliosphere may be calculated directly from the Boltzmann equation,

1 $$ \frac{\partial f }{\partial t} + {\mathbf{v}} \cdot \nabla f + \left ( \frac{{\mathbf{F}}}{m}\cdot \nabla _{ {\mathbf{v}} } \right ) f = P - L , $$

where $f( {\mathbf{x}} , {\mathbf{v}} , t)$ is the H or He particle distribution function expressed in terms of position ${\mathbf{x}} $, velocity ${\mathbf{v}} $ and time $t$. ${\mathbf{F}}$ is the force acting on a particle of mass $m$, typically gravity and radiation pressure. The terms $P$ and $L$ describe the production and loss of particles at $( {\mathbf{x}} , {\mathbf{v}} ,t)$, and both terms are functions of the assumed plasma and neutral distributions. In all cases of interest here, the loss term may be expressed as

2 $$ L = f( {\mathbf{x}} , {\mathbf{v}} ,t) \beta ( {\mathbf{x}} , {\mathbf{v}} ,t) , $$

where $\beta $ is the total loss rate in s^−1^. On defining the decay rate $\Lambda (t,t^{\prime} )$ as the loss of particles at a given location $( {\mathbf{x}} , {\mathbf{v}} )$ between times $t^{\prime}$ and $t$, one has

3 $$ \Lambda \left ( {\mathbf{x}} , {\mathbf{v}} ,t^{\prime} ,t \right ) \equiv \int _{t^{\prime }}^{t} \beta \left ( {\mathbf{x}} , {\mathbf{v}} , t^{\prime \prime } \right ) { \mathrm{d}}t^{\prime \prime }. $$

The formal solution to (1) for the initial data $f_{0} ( {\mathbf{x}} _{0} , {\mathbf{v}} _{0} ,t_{0})$ is then simply

4 $$ f( {\mathbf{x}} , {\mathbf{v}} ,t) = f_{0} ( {\mathbf{x}} _{0} , {\mathbf{v}} _{0} ,t_{0}) e^{-\Lambda (t_{0},t)} + \int _{t_{0}}^{t}P \left ( {\mathbf{x}} ^{\prime } , { \mathbf{v}} ^{\prime } ,t^{\prime } \right ) e^{-\Lambda (t^{\prime },t)} {\mathrm{d}}t^{\prime } . $$

The boundary data is assumed typically to be a Maxwellian distribution parameterized by the bulk LISM density, velocity and temperature and the boundary condition is imposed at “infinity”. Along the trajectory $( {\mathbf{x}} ^{\prime} , {\mathbf{v}} ^{\prime} , t^{\prime})$, neutral H atoms can experience the interactions/physical processes listed above using the cross sections listed in e.g., Table 1 of Zank (1999a). Simple and useful estimates for the production and loss of neutral H atoms were derived for charge-exchange production and loss by Ripken and Fahr (1983), forms of which are still used today (see e.g., Pauls et al. 1995). The neutral H loss rate due to charge-exchange is obtained by integrating over the proton distribution function, thus

5 $$ \beta _{ex} ( {\mathbf{x}} , {\mathbf{v}} ,t) = \int f_{p} ( {\mathbf{x}} , {\mathbf{v}} _{p} ,t) V_{rel,p} \sigma _{ex}(V_{rel,p}) {\mathrm{d}}^{3} {\mathbf{v}} _{p} , $$

where $f_{p}$ and ${\mathbf{v}} _{p}$ refer to proton quantities, $V_{rel,p} \equiv | {\mathbf{v}} - {\mathbf{v}} _{p} |$ is the relative speed between an H atom and a proton, and $\sigma _{ex}$ denotes the charge-exchange cross-section. If the proton distribution is cold with constant velocity ${\mathbf{v}} _{p, cold}$, i.e., if $f_{p} ( {\mathbf{x}} , {\mathbf{v}} ,t) = n_{p} ( {\mathbf{x}} ,t) \delta ^{3} ( { \mathbf{v}} - {\mathbf{v}} _{p, cold})$, then (5) reduces to

6 $$ \beta _{ex} ( {\mathbf{x}} , {\mathbf{v}} ,t) = n_{p} ( {\mathbf{x}} ,t) V_{rel,p} \sigma _{ex} (V_{rel,p}) , $$

and $V_{rel,p} \equiv | {\mathbf{v}} - {\mathbf{v}} _{p, cold} |$. The charge-exchange neutral hydrogen production term is given by

7 $$ P_{ex} ( {\mathbf{x}} , {\mathbf{v}} ,t) = f_{p} ( {\mathbf{x}} , {\mathbf{v}} _{H} ,t) \int f_{H} ( {\mathbf{x}} , {\mathbf{v}} ,t) V_{rel,H} \sigma _{ex} \left ( V_{rel, H} \right ) {\mathrm{d}}^{3} {\mathbf{v}} _{H} , $$

where $V_{rel,H} \equiv | {\mathbf{v}} - {\mathbf{v}} _{H} |$ and $f_{H}$ is the neutral H distribution. Typically, the plasma distribution $f_{p}$ is assumed to be a Maxwellian distribution although Malama et al. (2006) and Chalov et al. (2016) have considered more elaborate non-Maxwellian plasma distributions.

The so-called cold heliospheric neutral H and He model was extensively investigated in the late 1960’s and early 1970’s. The model continues to provide good physical insight but has since been overtaken by increasingly sophisticated models that assume a hot distribution of neutral H and He (Bzowski et al. 2012; Lee et al. 2012; Izmodenov et al. 2013). The cold models assume that the thermal speed of H or He is much less than its bulk flow speed relative to the Sun, which makes the solution of Boltzmann’s equation (1) particularly simple in the absence of production terms. The basic analysis was done by Fahr (1968), Blum and Fahr (1970), Holzer (1970), Holzer and Axford (1970, 1971) and summarized by Axford (1972). For a cold steady H distribution, subject to a spherically symmetric conservative potential

8 $$ F(r) = -\frac{G M_{\odot} (1 - \mu _{\odot} ) }{r} , $$

we seek a two-dimensional, axially symmetric solution to the Boltzmann equation (1). In (8), $r$ refers to heliocentric radius, $M_{\odot}$ to solar mass and $\mu _{\odot}$ is the ratio of radiation pressure to gravity. In equation (8), the solar radiation pressure has been approximated as a radial outward force that varies inversely with the square of distance from the Sun. This then yields the “effective” gravitational constant $(1 - \mu _{\odot} )G$. For H, the large solar Lyman-$\alpha $ flux corresponds to $\mu _{\odot} \simeq 1$. For heavy interstellar neutrals, such as He and O, $\mu _{\odot} \ll 1$ and can safely be neglected. The dynamical behavior of heavy atoms is therefore determined primarily by solar gravity, making the entrance of heavy atoms into the heliosphere essentially a problem of celestial mechanics (subject to losses). It was noted already by Axford (1972) that a more accurate treatment of the radiation pressure term would require the determination of the variation in $\mu _{\odot}$ as a consequence of both Doppler shifts and the reduction in intensity of the radiation due to scattering.

If we assume cold interstellar atoms, i.e., if the thermal velocity of the interstellar atoms is small compared to the bulk velocity of the gas relative to the Sun ($V_{\infty} \simeq 25~\text{km}\,\text{s}^{-1}$), then the trajectory of every atom lies in the plane determined by its velocity vector at infinity and the Sun. Since the atom-atom collisional mean free path is large and the most important collisions with solar wind particles are ionizing, we can treat the atoms as propagating freely in a gravitational potential subject to ionization losses.

**Fig. 1:**
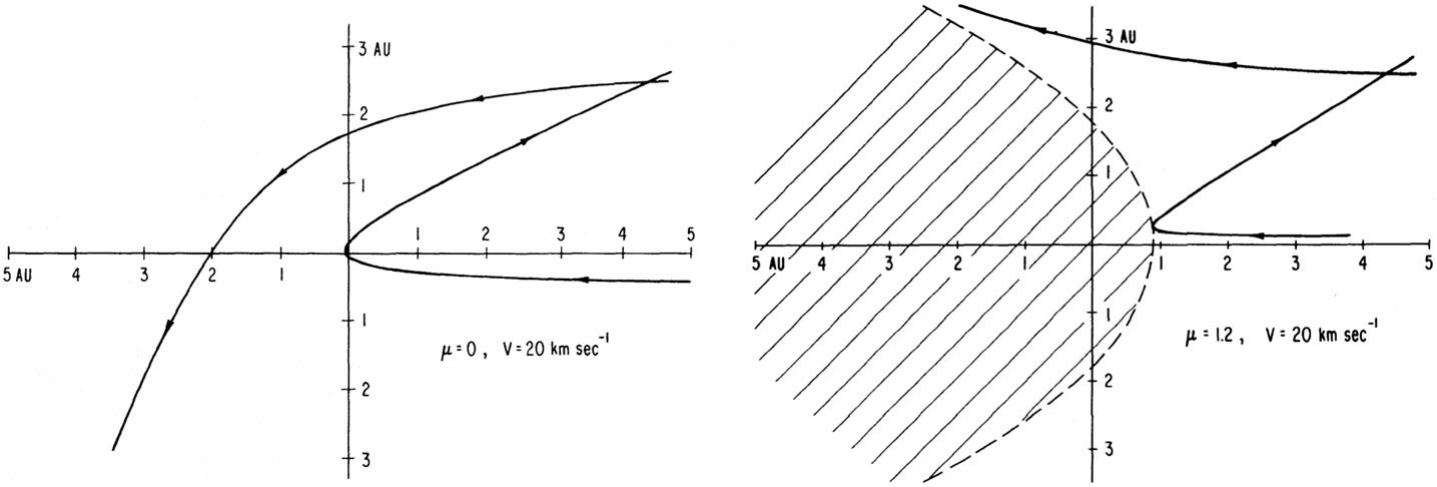
(Left) Two examples of intersecting particle trajectories in the cold interstellar H approximation in the absence of radiation pressure, i.e., $\mu _{\odot} = 0$. (Right) The same as (Left) but now with $\mu _{\odot} > 1$. The parabolic region downstream and about the Sun from which cold H atoms are excluded is hatched (Axford 1972)

Any point $(r,\theta ^{\prime})$ in the heliospheric plane is the intersection of two hyperbolic neutral particle trajectories having the Sun as focus (see Fig. 1). The cold neutral distribution at these points is therefore given by

9 $$ f_{H} ( {\mathbf{x}} , {\mathbf{v}} ) = n_{H,1} \delta ^{3} ( {\mathbf{v}} - {\mathbf{v}} _{H,1} ) + n_{H,2} \delta ^{3} ( {\mathbf{v}} - {\mathbf{v}} _{H,2} ) , $$

where $n_{H,i}$ refers to the neutral hydrogen number density and ${\mathbf{v}} _{H,i}$ to the velocity vectors. Any point in the plane $(r,\theta ^{\prime})$ is the intersection of two trajectories (illustrated in Fig. 1) having angular momentum $p_{\pm}$ per unit mass or impact parameter $b_{\pm}$

10 $$\begin{aligned} b_{\pm} \equiv \frac{p_{\pm} }{V_{\infty}} = \frac{1}{2} \left \{ r \sin \theta ^{\prime} \pm \left [ r^{2} \sin ^{2} \theta ^{\prime} + 4 r \left ( 1 - \mu _{\odot} \right ) \frac{GM_{\odot}}{V_{\infty}^{2}} \left ( 1 - \cos \theta ^{\prime} \right ) \right ]^{1/2} \right \} . \end{aligned}$$

The shorter of the paths is often referred to as the direct path, orbit, or trajectory and the longer path that grazes the Sun is called the indirect path, orbit, or trajectory.

Illustrated in Fig. 1 is the motion of a single (H) atom subject to the potential (8). As illustrated, $p_{+} > 0$, $p_{-} < 0$ if $\mu _{\odot} < 1$, and $p_{+} > 0$, $p_{-} > 0$ if $\mu _{\odot} > 1$. Thus, Fig. 1 (left) shows a representative single particle trajectory with a dominant gravitational force ($\mu _{ \odot} < 1$) and Fig. 1 (right) that of a dominant solar Lyman-$\alpha $ radiation pressure ($\mu _{\odot} > 1$). For $\mu _{\odot} <1$, one can expect that H atoms are focussed on the downward symmetry axis. For $\mu _{\odot} < 1$, some particles may be accreted onto the Sun, whereas for $\mu _{\odot} > 1$, the particle stream cannot enter a region defined by

11 $$ r (1 + \cos \theta ^{\prime} ) \leq \left ( \frac{4GM}{V_{\infty}^{2}} \right ) (\mu _{\odot} - 1) , $$

since the angular momentum (10) becomes imaginary. For $\mu _{\odot} = 1$, atom trajectories are obviously straight lines parallel to $\theta ^{\prime} = 0$ and $p_{-} = 0$. The number density $n_{a}$ of interstellar atoms $a$ in the solar wind can be computed from the continuity equation using the neutral atom trajectories as streamlines. This then yields

12 $$ n_{a}(r,\theta ) = n_{a0} \frac{b_{+}^{2} \exp \left [ -\beta r^{2} \theta /p_{+} \right ] + b_{-}^{2} \exp \left [ -\beta r^{2} \left ( 2 \pi - \theta \right ) /p_{-} \right ] }{ r\sin \theta \left [ r^{2} \sin ^{2} \theta + 4r \left ( 1 - \cos \theta \right ) \left (1 - \mu _{\odot} \right ) GM_{\odot} /V_{\infty}^{2} \right ]^{1/2} } . $$

Here $n_{a0}$ is the number density of the neutral particles at infinity, and $\theta $ is the polar angle from the axis of symmetry ($0 \leq \theta \leq \pi $ and $|\sin \theta ^{\prime} | = \sin \theta $). In the limit that radiation pressure balances gravitational attraction exactly, i.e., that $F(r)= 0$, then $v = V$, $b_{+} = Vr \sin \theta ^{\prime}$, $p_{-} = 0$, and expression (12) reduces to

13 $$ n_{a} (r, \theta ) = n_{a0} \exp \left [ - \frac{\beta r \theta }{V_{\infty} \sin \theta } \right ]\equiv n_{a0} \exp \left [ -\frac{ \lambda \theta }{r \sin \theta} \right ], $$

after assuming that $\beta = \beta _{0} r_{0}^{2} /r^{2}$, and introducing $\lambda \equiv \beta _{0} r_{0}^{2} /V_{\infty}$.

Under the assumption of a spherically symmetric and steady solar wind and solar radiation field, $\beta _{0} r_{0}^{2}$ is independent of $r_{0}$ (at least if one has a minimal attenuation of the photon flux and if one ignores the accretion of interstellar protons), and the interstellar neutral hydrogen population is strongly depleted within some 6–10 AU. This region of depleted interstellar neutral hydrogen is called the ionization cavity. The effect of focussing and exclusion on the heliospheric distribution of interstellar hydrogen is described by (12). If one plots contours of equal density for neutral H for various values of $\mu _{\odot}$, one finds a high density on the downstream axis of symmetry when $\mu _{\odot} < 1$ and a parabolic void when $\mu _{\odot} > 1$ (Axford 1972).

Although a useful approximation that provides considerable insight, the assumed cold H and He distributions are not completely adequate in that (i) LISM thermal speeds and bulk flow speeds are comparable, and (ii) the LISM temperature may be estimated for heliospheric resonance observations only if it is included as a model parameter. Thus, considerable efforts have been expended in extending the cold heliospheric neutral hydrogen and helium models to an initial interstellar Maxwellian distribution, historically these being Danby and Camm (1957), Fahr (1971), Thomas and Krassa (1971), Fahr (1979), Wu and Judge (1979, 1980), and useful reviews were provided by Meier (1977) and Thomas (1978).

Considerable impetus to revisit the hot models and extend the modeling efforts, particularly for neutral He, was provided by the IBEX mission. The IBEX-Hi and IBEX-Lo telescopes measure energetic neutral atoms (ENAs) at 1 AU. However, IBEX-Lo can measure part of the neutral interstellar gas distribution directly, allowing us to infer properties of the interstellar parent populations via backward modeling from the observed neutral gas distributions.

Assumptions very similar to those made for the cold model are made again for the hot distribution except that now the source distribution function is assumed to be a Maxwellian, i.e.,

14 $$ f_{0} ({\mathbf{x}}_{0} , {\mathbf{v}}_{0} ) = \frac{n_{\infty} }{ ( \sqrt{\pi} v_{th, \infty} )^{3} } \exp \left [ - \left ( \frac{ {\mathbf{v}}_{0} - {\mathbf{u}} }{ v_{th, \infty} }\right )^{2} \right ] , $$

where now ${\mathbf{u}}$ is the bulk neutral flow speed at infinity rather than the $V$ of the cold model section. Use of (14) in the formal solution (4) without the production term yields the distribution function

15 $$ f ( {\mathbf{x}}, {\mathbf{v}} ) = \frac{n_{\infty} }{ ( \sqrt{\pi} v_{th, \infty} )^{3} } \exp \left [ - \left ( \frac{ {\mathbf{v}}_{0} - {\mathbf{u}} }{ v_{th, \infty} }\right )^{2} \right ] \exp \left [ - \Lambda \right ] H\left ( v_{0}^{2} \right ), $$

where

$$ v_{0}^{2} \equiv v^{2} - \frac{2GM_{\odot} ( 1 - \mu _{\odot} ) }{r} , $$

and $H(x)$ is the usual Heaviside step function. By solving Kepler’s equation for the neutral trajectories, one has

16 $$ \left ( \frac{ {\mathbf{v}}_{0} - {\mathbf{u}} }{ v_{th, \infty} } \right )^{2} = \frac{1}{ \left ( v_{th, \infty} \right )^{2} } \left [ v_{0}^{2} + u^{2} + 2v_{0} u \left ( \frac{v_{z} (v_{0} - v_{r} ) - F(r) \cos \theta }{v_{0} (v_{0} - v_{r} ) - F(r) } \right ) \right ], $$

where $v_{r}$ and $v_{z}$ are the radial and $z$ direction components of the velocity vector ${\mathbf{v}}$ and $F(r)$ is the potential (8). If one assumes again that $\beta = \beta _{0} r_{0}^{2}/r^{2}$, then

17 $$ \Lambda = \frac{\beta _{0} r_{0}^{2} \theta ^{\prime} }{v_{0} p_{0} } , $$

where $\theta ^{\prime}$ is the angle swept out by the atom on its Keplerian trajectory and $p_{0} = |{\mathbf{r}} \times {\mathbf{v}} |$ is the angular momentum. In the limit $v_{th, \infty} \rightarrow 0$, the hot distribution function reduces to the cold expression (9) except on the LISM flow axis and in the forbidden region (11). The number density, velocity and temperature for the hot distribution can be obtained from (15) by taking appropriate moments, a process which is essentially numerical, although some analytic approximations can be made (Danby and Bray 1967; Wu and Judge 1979). Several important points emerge from numerical solutions of the hot model for the distribution of hydrogen and helium in the heliosphere. (i) The neutral radial velocity distribution $N({\mathbf{r}}, v_{r})$, ${\mathbf{r}}$ the spatial position and $v_{r}$ the radial velocity, at 1 AU is very well fitted by a Maxwellian distribution (Wu and Judge 1979), as is that for interstellar He at 1 AU. For $\theta \geq 90^{\circ}$, He atoms with both direct and indirect trajectories contribute to the neutral density, and the total distribution is described by two superimposed Maxwell-Boltzmann distributions with different temperatures. For $\theta < 90^{\circ}$, the family of indirect orbits correspond to large angular variations and the contribution from these atoms is virtually negligible. Thus, for $\theta < 90^{\circ}$, the interstellar He velocity distribution is essentially a single peaked velocity distribution. In the downwind region, $\theta > 150^{\circ}$, the contributions of the direct and indirect He distributions merge, producing an approximately single Maxwellian distribution. (ii) An asymmetry in the heliospheric neutral H temperature gradient was predicted. For upwind directions, the H temperature decreases with decreasing heliocentric distance, whereas the opposite is true for the downwind direction. As discussed by Wu and Judge (1979), the H temperature increase downwind is due primarily to ionization losses. For He, the weak ionization loss process does not modify strongly the He temperature, but since gravitational focussing now dominates, the He temperature can be significantly modified. The He temperature in the upwind direction decreases with decreasing heliocentric distance. However, the He temperature is largest at $\theta = 150^{\circ}$ rather than directly downwind ($\theta = 180^{\circ}$) since the contribution to the He density by atoms following indirect orbits is largest in this region of phase space, and thus causes a significant velocity spread and therefore an effective temperature increase. Far from the downwind region, as discussed above, the direct and indirect trajectories of He atoms do not merge and relatively distinct distributions, and thus distinct temperatures, are required to describe the He temperature. The temperature of the He distribution associated with indirect orbits is always less than the temperature associated with direct orbits. (iii) An ionization cavity is evident within 6–10 AU and the cavity is elongated in the downstream direction. For $\mu _{\odot} > 1$, the downstream region is further depleted. Nonetheless, the downstream singularity of the cold model is eliminated by a hot neutral distribution, as is the paraboloid void when $\mu _{\odot} > 1$. However, these regions continue to posses the basic characteristics of the cold model. (iv) The interplanetary H velocity for $\mu _{\odot} > 1$ decreases with decreasing heliocentric distance and the hot model produces slightly lower speeds than the cold model for $v > 0$ and higher for $v < 0$. The radial velocity of He is independent of the interstellar temperature except marginally at $\theta \simeq 180^{\circ}$. Thus, solar gravitational focussing is the primary process that modifies the bulk velocity of inflowing neutral He, and the assumed interstellar temperature of neutral He plays almost no role in the radial velocity of He within the heliosphere. Furthermore, the low ionization loss rate of He leads to little change in the He radial velocity.

### The Creation of Pickup Ions in the Solar Wind and Their Properties

Interstellar neutral gas flows relatively unimpeded into the heliosphere, certain species of which experience some “filtration” at the heliospheric boundaries. Neutral interstellar hydrogen is especially susceptible to the effects of filtration, being decelerated and heated in passing from the LISM into the heliosphere. The interstellar neutral gas flowing into the supersonic solar wind can be ionized by either solar photons (photoionization) or solar particles (charge exchange, electron-impact ionization) and the new ions respond almost instantaneously to the electromagnetic fields of the solar wind. In the solar wind frame of reference, the newly born interstellar ions immediately gyrate about the interplanetary magnetic field (IMF), after which they experience scattering and isotropization by either ambient or self-generated low-frequency electromagnetic fluctuations in the solar wind plasma. Since the newly born ions are eventually isotropized, their bulk velocity is now that of the solar wind i.e., they are advected with the solar wind flow, and are then said to be “picked up” by the solar wind. The isotropized pickup ions (PUIs) form a distinct population of energetic ions ($\sim 1~\text{keV}$) in the supersonic solar wind whose origin is the interstellar medium. Similarly, pickup ions can be created in the inner heliosheath by charge-exchange with inflowing interstellar neutral atoms or outflowing neutral atoms created in the supersonic solar wind, or even in the interstellar medium, although the importance of this has only been recognized since 1995. Consequently, we focus only on PUIs in the supersonic solar wind.

Since the neutral interstellar hydrogen gas flows into the heliosphere at $\sim 20~\text{km}/\text{s}$ (see later chapters discussing global models of the solar wind – interstellar medium interaction), it is supersonic in the solar wind frame. Newly created pickup ions can therefore drive a host of plasma instabilities. A newly ionized pickup ion is accelerated immediately by the motional solar wind electric field ${\mathbf{E}} = - {\mathbf{u}} \times {\mathbf{B}}$, where ${\mathbf{u}}$ is the solar wind flow velocity and ${\mathbf{B}}$ the ambient IMF. In a Cartesian frame co-moving with the solar wind, the velocity of a pickup ion is simply ${\mathbf{v}} (t) = \left (-u_{\perp} \cos \Omega _{i} t , u_{\perp} \sin \Omega _{i} t , u_{\parallel}\right )$, where the IMF is oriented along $\hat{\mathbf{z}}$, $u_{\parallel}$ is parallel to $\hat{\mathbf{z}}$, $u_{\perp}$ is perpendicular to $\hat{\mathbf{z}}$, and $\Omega _{i} \equiv q B/m$ is the local pickup ion gyrofrequency ($q$ denoting charge and $m$ ion mass). The pickup ions therefore form a ring-beam distribution on the time scale $\Omega _{i}^{-1}$ which streams sunward along the magnetic field.

Both the anisotropy of the ring-beam distribution and its relative streaming with the solar wind drive instabilities that remove energy from the distribution and excite waves. Wu and his colleagues (Wu and Davidson 1972; Wu et al. 1973; Hartle and Wu 1973; Wu and Hartle 1974; Wu et al. 1986) used an idealized narrow ring-beam distribution (i.e., the pickup ions all have identical speed and pitch-angle) to show that hydromagnetic and whistler modes propagating parallel to ${\mathbf{B}}$ become unstable. The instability analysis of Wu and Davidson (1972) has been generalized and extended by several authors, primarily in the context of cometary pickup ions (Winske et al. 1985; Winske and Gary 1986; Sharma and Patel 1986; Brinca and Tsurutani 1988; Gary et al. 1988; Gary and Madland 1988). It was pointed out by Lee and Ip (1987) that the assumption of a sharp narrow ring-beam distribution was not warranted and they determined maximum growth rates for a broad ring-beam distribution. Other instabilities, such as the firehose instability and a whistler instability were considered by Wu and Davidson (1972) on the basis of the ring-beam distribution. The latter instability is however significantly reduced when a broad ring-beam distribution is assumed (Lee and Ip 1987).

**Fig. 2:**
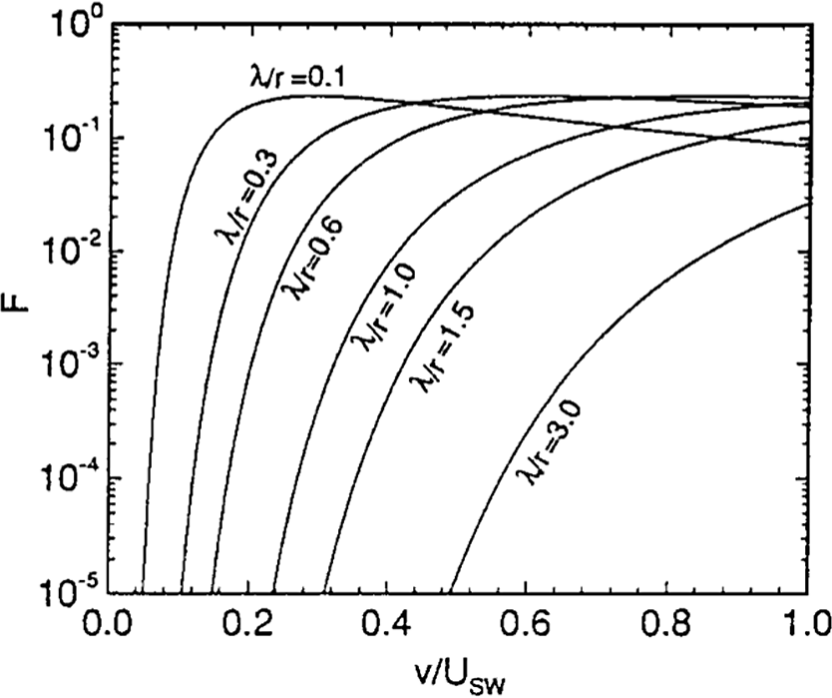
The velocity distribution function for interstellar ions (20) using the cold hydrogen distribution (Zank 1999a)

Vasyliunas and Siscoe (1976) investigated the evolution of the pickup ion distribution in the absence of energy diffusion. Isenberg (1987) generalized this calculation by including energy diffusion. In a steady, spherically symmetric expanding solar wind, an isotropic distribution of pickup ions evolves as

18 $$ \frac{\partial f}{\partial t} + u \frac{\partial f}{\partial r} - \frac{2u }{r}\frac{v}{3} \frac{\partial f}{\partial v} = \frac{1}{v^{2}} \frac{\partial}{\partial v} \left ( v^{2} D \frac{\partial f}{\partial v} \right ) + \frac{n_{H}}{\tau _{ion} } \frac{ \delta (v - u) }{4\pi v^{2}} , $$

where $n_{H} = n_{H}^{\infty} \exp \left [ -\lambda \theta / r \sin \theta \right ]$ and $\tau _{ion} = \tau _{ion}^{0} r^{2}/r_{0}^{2}$ (Vasyliunas and Siscoe 1976; Isenberg 1987). In (18), it has been assumed that the isotropization of the initial ring-beam distribution is immediate so that the source term may be approximated as an isotropic shell moving at the solar wind speed. Equation (18) is solved easily using the method of characteristics in the limit that $D = 0$. In this case, the steady-state solution is given by

19 $$ f(r,v) = \frac{3}{8\pi} \frac{n_{H} (r_{1}) }{\tau _{ion}^{0} u ^{2} } \frac{r_{0}^{2}}{r} \left ( \frac{u }{v} \right )^{3/2} , $$

where $r_{1} = \left ( r^{2} v^{3} / u ^{3} \right )^{1/2}$. For the simple cold distribution, (19) reduces to (Vasyliunas and Siscoe 1976)

20 $$ f(r,v) = \frac{3}{8\pi} \frac{n_{H}^{\infty} }{\tau _{ion}^{0} u ^{2} } \frac{r_{0}^{2}}{r} \left ( \frac{u }{v} \right )^{3/2} \exp \left [ \frac{-\lambda}{r} \frac{\theta}{\sin \theta} \left ( \frac{u }{v} \right )^{3/2} \right ] , $$

illustrated in Fig. 2. Somewhat serendipitously, interstellar $\text{He}^{+}$ PUIs were discovered with the SULEICA instrument on AMPTE IRM (Moebius et al. 1985) as a result of the first AMPTE lithium ion release into the solar wind (Möbius et al. 1986). It is not surprising that $\text{He}^{+}$ PUIs were observed first because He has the highest ionization potential of all elements. Thus, most interstellar He survives to 1 AU, making it the dominant interstellar species at 1 AU. The PUI $\text{He}^{+}$ spectral observations were shown to provide a reasonable quantitative match with a simple model in Moebius et al. (1988). These results were then used to provide the first determination of the interstellar He flow parameters and temperature from *in situ* measurements of the He focusing cone (Moebius et al. 1995).

**Fig. 3:**
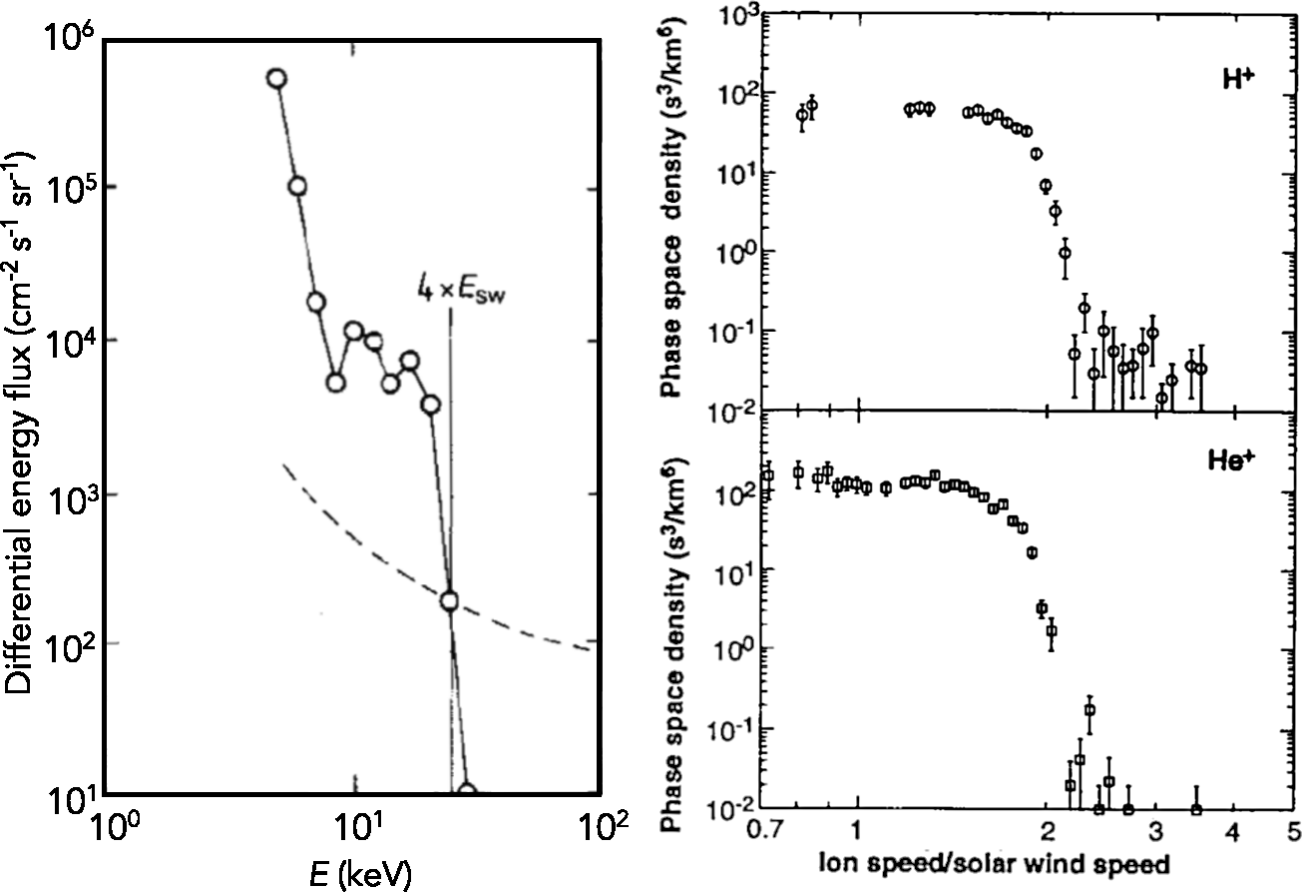
(Left) Differential energy flux spectrum of $\text{He}^{+}$ PUIs taken observed with AMPTE SULEICA on Nov 11, 1984. A plateau and the PUI cut-off at $2V_{sw}$ ($4E_{sw}$ for $\text{He}^{+}$) are clearly visible. The rise at $\simeq 5\text{ keV}$ is due to the presence of heavy solar wind ions. The dashed line indicates the 1-count level (Moebius et al. 1985). (Right) The phase space density of interstellar pickup protons (top) and $\text{He}^{+}$ (bottom) as a function of $v/u$ in the spacecraft frame observed by the SWICS instrument on the Ulysses spacecraft at 4.82 AU (Gloeckler et al. 1993)

**Fig. 4:**
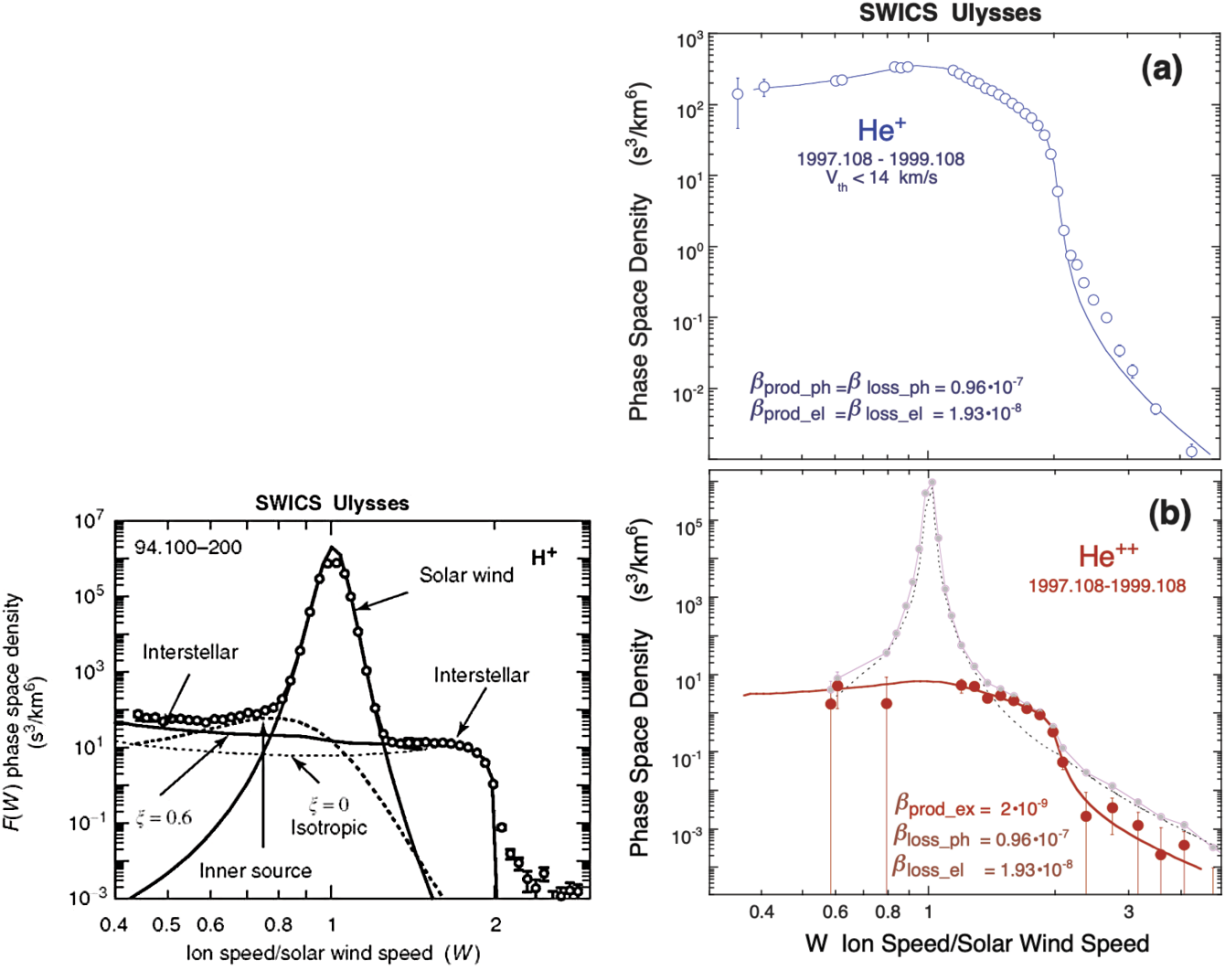
(Left) The combined velocity distribution function for solar wind and interstellar pickup $\text{H}^{+}$ as a function of $W = v/u$ in the spacecraft frame observed by the SWICS instrument on the Ulysses spacecraft at 4.82 AU. The solar wind $\text{H}^{+}$ distribution corresponds to a Maxwellian distribution function, and the presence of the flat-topped PUI distribution is clearly evident at suprathermal energies (Gloeckler et al. 1993, 1994b). (Right) The combined velocity distribution function for solar wind and interstellar pickup $\text{He}^{+}$ (upper panel, open circles) and $\text{He}^{++}$ (lower panel, solid circles) as a function of $W \equiv v/u$ in the spacecraft frame observed by the SWICS instrument on the Ulysses spacecraft. Model curves are computed using ionization rates, $\beta $, given next to each model curve and interstellar parameters given in Gloeckler et al. (2004) (see Gloeckler et al. (2004) for more details). The $\text{He}^{+}$ velocity spectrum shows the characteristic sharp cutoff near $W = 2$, and a suprathermal tail at higher speeds. The best fit requires a density $n_{He} = 0.016~\text{cm}^{-3}$ for neutral helium near the heliospheric termination shock at around 100 AU. The pickup $\text{He}^{++}$ distribution (lower panel, solid bold circles) was obtained from the measured total $\text{He}^{++}$ spectrum (solar wind plus pickup $\text{He}^{++}$, solid faint circles) by subtracting from it the solar wind distribution (dotted curve). Pickup $\text{He}^{++}$ is produced primarily by charge exchange with solar wind alpha particles (Gloeckler et al. 2004)

Ulysses observed multiple interstellar pickup ion species, including the first measurements of interstellar pickup H, an extensive summary of which can be found in the reviews by Gloeckler et al. (1994b, 2004). Plots of $f(r,v)$ using (20) for different values of $\lambda /r$ are illustrated in Fig. 3a and they show that, with increasing heliocentric distance, the velocity distribution becomes increasingly flat-topped. In Fig. 3b, a phase-space plot (in the spacecraft frame) of pickup ions ($\text{H}^{+}$ and $\text{He}^{+}$) observed by ULYSSES is shown (Gloeckler et al. 1993). The sharp cutoff at $v/u = 2$ is clearly evident. A corresponding total distribution that includes both solar wind and pickup $\text{H}^{+}$ is illustrated in the left panel of Fig. 4. The solar wind distribution is well described by a Maxwellian, and the PUIs have a flat-topped distribution form until about twice the solar wind speed followed by a suprathermal tail. The right panel of Fig. 4 shows pickup $\text{He}^{+}$ and $\text{He}^{++}$ in the solar wind. Like $\text{H}^{+}$, the $\text{He}^{+}$ velocity spectrum shows the characteristic sharp cutoff near $W = v/u = 2$, and a suprathermal tail at higher speeds. The bottom panel shows pickup $\text{He}^{++}$, which is a consequence of charge exchange with solar wind alpha particles. The pickup $\text{He}^{++}$ distribution function exhibits the same characteristics as the pickup $\text{He}^{+}$ distribution.

The sharp cutoff in the pickup ion distribution (in the solar wind rest frame) indicates that adiabatic cooling dominates energy diffusion.

Computer simulations of ion pickup in the supersonic solar wind proved instructive in these early studies. For $0^{\circ} < \theta \leq 45^{\circ}$, the ion-ion right-hand resonant instability is the dominant mode, and for $\theta \geq 70^{\circ}$, the left-hand polarized ion cyclotron instability begins to dominate (Brinca and Tsurutani 1988; Gary et al. 1989; Gary 1991). The ion-ion right-hand resonant instability growth rate is greatest for waves parallel to ${\mathbf{B}}$ and decreases monotonically with increasing obliquity. For relatively small ion drift velocities parallel to the magnetic field, the growth of waves can be quite localized along the field. In these cases, the ion-ion non-resonant instability may dominate. Computer simulations by Gary and Winske (1990) show that the ion-ion anisotropy instability saturates sooner than the ion-ion beam instability. Other simulations (Gary et al. 1989) show a decreasing level of fluctuations as $\theta $ increases; a result which is consistent with observations made at comet Giacobini-Zinner (Tsurutani and Smith 1986a,b) which show the absence of large amplitude magnetic fluctuations near the water group ion cyclotron frequency when $\theta \simeq 90^{\circ}$. The basic conclusion that emerges from simulations is that the pickup of interstellar atoms should drive the fastest growing, largest amplitude waves when the IMF is aligned almost radially with the solar wind flow. By contrast, the waves should saturate at a low level in regions where $\theta \simeq 90^{\circ}$.

Because the pickup process is predicted to generate substantial wave activity as the unstable ring-beam distribution is isotropized, evidence for these waves in the solar wind magnetic field data measured by Voyager 1 and 2 and Ulysses was expected to be found easily in the outer heliosphere beyond the ionization cavity, e.g., Lee and Ip (1987), Williams and Zank (1994), Isenberg and Lee (1996). Spectral enhancements were predicted to appear at spacecraft-frame frequencies greater than, but comparable to, the proton cyclotron frequency $\Omega _{p} = eB/m_{p}$. However, PUI-generated waves have been very difficult to observed despite their predicted ubiquity (Lee and Ip 1987). Murphy et al. (1995) presented a preliminary survey of 31 cases in the Ulysses data during a 640 day interval around 5 AU when the spacecraft was in the neighborhood of Jupiter, i.e., low-latitude observations at the extreme range of the Ulysses trajectory. Waves were seen at the expected spacecraft-frame frequencies greater than, but comparable to, $\Omega _{p}$, which was taken as evidence of wave excitation by pickup protons.

Consider now the evolution of the PUI distribution in the supersonic solar wind as it is scattered in pitch-angle by magnetic field fluctuations, both those generated by the pickup ions themselves as well as preexisting *in situ* turbulence. A number of processes determine the evolution of the pickup ion distribution – primarily pitch-angle scattering and energy diffusion in the wave and/or turbulence field, convection and adiabatic deceleration in the expanding solar wind, and the injection of newly ionized particles. These various processes all possess different time-scales, and pitch-angle scattering should dominate due to the large growth rate of the unstable waves and the high pickup ion velocities ($v \sim u$, the solar wind speed). Since $|V_{A}| \ll v$, classical energy diffusion is unlikely to be an important factor in determining the gross evolution of the pickup ion distribution.

### The Pickup Ion-Mediated Supersonic Solar Wind

Although number densities are too low for the direct interaction of the solar wind plasma flow with the neutral flux, appreciable momentum and energy exchange is possible nonetheless through charge exchange of solar wind protons and neutral hydrogen. Although the microscopic details of this process are complicated and depend on the plasma-magnetic field configuration, the net result of ion pickup on hydrodynamic scales is qualitatively unique – there is a change in the density, momentum, and energy of the plasma flow for each act of charged particle production or destruction. The basic solar wind models that incorporated pickup ions at some level of consistency were formulated in the seminal and far-reaching papers of Wallis (1971) and Holzer (1972), building on earlier work (Axford et al. 1963; Patterson et al. 1963; Dessler 1967; Hundhausen 1968; Fahr 1968; Blum and Fahr 1970; Semar 1970; Holzer and Axford 1970).

The first models of the supersonic solar wind mediated by pickup ions did not distinguish between pickup protons and solar wind protons. We consider the simpler models that treated the solar wind plasma as a single fluid. Khabibrakhmanov et al. (1996) formalized the one-fluid models of Wallis (1971) and Holzer (1972). The magnetic field can be neglected (although see Holzer 1972 for its inclusion) to leading order in the outer heliosphere since the magnetic pressure is small compared to that of the total thermal pressure $P$ when the PUI contribution is included and the solar wind ram pressure. On assuming spherical symmetry, the hydrodynamic one-fluid model may therefore be expressed as (Wallis 1971; Holzer 1972; Khabibrakhmanov et al. 1996);

21 $$\begin{aligned} n_{t} + \frac{1}{r^{2}} \left ( r^{2} u n \right )_{r} &= \nu _{ph} N ; \end{aligned}$$

22 $$\begin{aligned} \rho u_{t} + \rho u u_{r} + P_{r} &= - \nu _{ph} m Nu - \langle \sigma _{c} v \rangle N \rho u ; \end{aligned}$$

23 $$\begin{aligned} P_{t} + uP_{r} + \gamma Pu_{r} + \frac{2}{r} \gamma u P &= (\gamma - 1) \nu _{ph} mN\frac{u^{2}}{2} \\ &\quad{}- \langle \sigma _{c} v \rangle N \left [ P - (\gamma - 1) \rho \frac{u^{2}}{2} \right ] . \end{aligned}$$

The geometry of the pickup ion interaction and its physical properties are determined by the angle between the magnetic field direction and the flow direction. In the case that the magnetic field is orthogonal to both the plasma flow and the neutral H flow, then newly born ions acquire motion only in the plane orthogonal to the magnetic field direction. Thus, in this configuration, newly born ions have 2 degrees of freedom, implying that the pickup ions behave on hydrodynamic scales as a gas with specific heat ratio $\gamma = 2$. However, we expect the magnetic field and the neutral H flux are not orthogonal and therefore have an additional degree or freedom, and critically, there is bulk motion of the new born ions with respect to the rest of the plasma. As discussed, the distribution is unstable and rapidly isotropizes almost completely, yielding a perfect gas with $\gamma = 5/3$. Hence, for quasi-perpendicular geometries, $\gamma = 2$, whereas $\gamma = 5/3$ for oblique and parallel geometries. Equations (22) and (23) can be expressed in conservation form. If we restrict our attention to the supersonic solar wind and neglect the thermal motion of plasma particles, one can assume that the charge exchange cross section is independent of velocity and use the approximation

$$ \langle \sigma _{c} v \rangle = \sigma _{c} u. $$

The momentum and energy equations are then

24 $$\begin{aligned} &\left ( \rho u \right )_{t} + \frac{1}{r^{2}} \left ( r^{2} \rho u^{2} \right )_{r} + P_{r} = -\sigma _{c} N \rho u^{2} ; \end{aligned}$$

25 $$\begin{aligned} &\left ( \frac{\rho u^{2}}{2} + \frac{1}{\gamma - 1} P \right )_{t} + \frac{1}{r^{2}}\left [ r^{2} u \left ( \frac{\rho u^{2}}{2} + \frac{\gamma}{\gamma - 1} P \right )\right ]_{r} \\ &\quad{} = -\sigma _{c} Nu \left ( \frac{1}{\gamma - 1} P + \frac{\rho u^{2}}{2}\right ) . \end{aligned}$$

The neutral gas density in the supersonic solar wind can be found in a crudely self-consistent fashion. For a given neutral gas flux $N_{\infty} V_{\infty}$ at infinity (in practice, because of filtration at the heliospheric boundaries, this should be the location of the heliopause), the neutral gas number density along the stagnation line can be obtained from the neutral gas continuity equation,

26 $$ -\frac{{\mathrm{d}} (VN) }{ {\mathrm{d}} r} = -\nu _{ph} N - \langle \sigma _{c} v \rangle Nn . $$

The two continuity equations (21) and (26) can be combined as a single second-order differential equation after assuming a fixed velocity for the neutral interstellar gas $V_{\infty}$, thus

27 $$ \frac{{\mathrm{d}} }{{\mathrm{d}} r}\left ( \frac{r^{2}}{N} \frac{{\mathrm{d}} N}{{\mathrm{d}} r} \right ) = \frac{\sigma _{c} \nu _{ph} N}{V_{\infty} } , $$

subject to the boundary conditions

28 $$ N(\infty ) = N_{\infty} ; \qquad \frac{V_{\infty} }{N} \frac{{\mathrm{d}} N}{ {\mathrm{d}} r} (r = r_{0} ) = \nu _{ph}^{0} + \sigma _{c} n_{0} u_{0} , $$

where “0” denotes evaluation at 1 AU. The transformation

$$ N/N_{\infty} = \exp \left [ \frac{ \nu _{ph}^{0} + \sigma _{c} n_{0} u_{0} }{V_{\infty} r /r_{0}} + y(x) \right ] , \qquad x \equiv - r_{0} / r , $$

reduces (27) to an equation in $y(x)$ with homogeneous boundary conditions

29 $$ x^{2} \frac{ {\mathrm{d}}^{2} y }{ {\mathrm{d}} x^{2}} = \frac{\sigma _{c} \nu _{ph} }{V_{\infty} }\exp \left [ V_{\infty}^{-1} (\nu _{ph}^{0} + \sigma _{c} n_{0} u_{0} ) x + y(x) \right ] , \;y(0) = 0 ; \: y^{\prime} (-1) = 0 . $$

By regarding the term $\sigma _{c} \nu _{ph} / V_{\infty}$ as a small parameter, the zeroth-order solution to (29) is the familiar result

$$ N/N_{\infty} = \exp \left [ \frac{ \nu _{ph}^{0} + \sigma _{c} n_{0} u_{0} }{V_{\infty}^{2} r /r_{0}^{2}} \right ] , $$

(Axford 1972; Vasyliunas and Siscoe 1976). By introducing the sound speed $C_{s}^{2} = \gamma P/\rho $ and the Mach number $M =u/c$, Eqs. (21)–(26) can be combined as an equation for $M^{2}$ (Wallis 1971; Holzer 1972; Khabibrakhmanov et al. 1996)

30 $$\begin{aligned} \frac{M^{2} - 1}{M^{2}} \frac{ {\mathrm{d}} M^{2}}{ {\mathrm{d}} r} &= \frac{2}{r} \left [ 2 +(\gamma - 1)M^{2} \right ] \\ &\quad{}+ \sigma _{c} N \frac{\gamma + 1}{\gamma} - \frac{N}{2}\left ( \sigma _{c} + \frac{m\nu _{ph} }{r^{2} \rho u} \right ) \left ( \gamma M^{2} + 1\right ) \left [ (\gamma - 1)M^{2} + 2 \right ] . \end{aligned}$$

Expression (30) is a little less general than that given by Holzer (1972) since magnetic and gravitational terms are neglected here but all the important points can nonetheless be made on the basis of this equation.

A closed form solution to the wind equation (30) cannot be obtained but general properties are easily inferred and numerical solutions are straightforward to obtain. The spherical expansion of the solar wind introduces the possibility of a smooth transition from a supersonic to a subsonic flow. A related extensive discussion of the deceleration of the solar wind in the vicinity of an outgassing comet also exists (e.g., Ip and Axford 1990). The critical point at $M = 1$ (when both the left-hand side and right-hand side of (30) are zero simultaneously) is quite different from that of the familiar critical point that arises in Parker’s model of the expanding and accelerating solar wind (Parker 1958; Holzer 1979). The sonic point near the sun is a saddle point with only one physically meaningful solution passing along a separatrix through the critical point. The topology of the steady-state solutions near the critical point admitted by (30) is more complicated. Beyond the ionization cavity, charge exchange is sufficiently effective to both decelerate the flow and, more importantly, to increase the effective solar wind “temperature” (by including the hot pickup ion halo). The net effect is to decrease the solar wind Mach number. The radial Mach number profiles are all asymptotic to the same value as $r$ increases. Such behavior can be understood in terms of the nature of the critical point which exists at large heliocentric distances as the Mach number approaches 1. The critical or sonic point is an improper node with two separatices. The physically meaningful upstream solutions all approach the critical point along the lower separatrix and the low Mach number solutions are indistinguishable from the separatrix. This mathematical possibility set off a prolonged and sometimes contentious debate about whether a discontinuous (i.e., a heliospheric termination shock) or smooth transition should decelerate the distant solar wind. Observationally, of course, Voyager 1 & 2 have confirmed the existence of the HTS rather than a smooth transition. A related debate has had a more complicated outcome in the context of the deceleration of the solar wind in the vicinity of comets with varying outgassing strengths (Ip and Axford 1990). If we assume that the critical Mach number $M_{c}$ of the termination shock is determined by the internal stability of the mass-, momentum-, and energy-loaded solar wind flow (Khabibrakhmanov et al. 1996), then the distance to the termination shock is determined by the interstellar neutral gas density $N^{\infty}$ only. Such a criterion is quite different conceptually from the usual manner in which the termination shock is located i.e., a balancing of the forces between the solar wind and the interstellar medium.

**Fig. 5:**
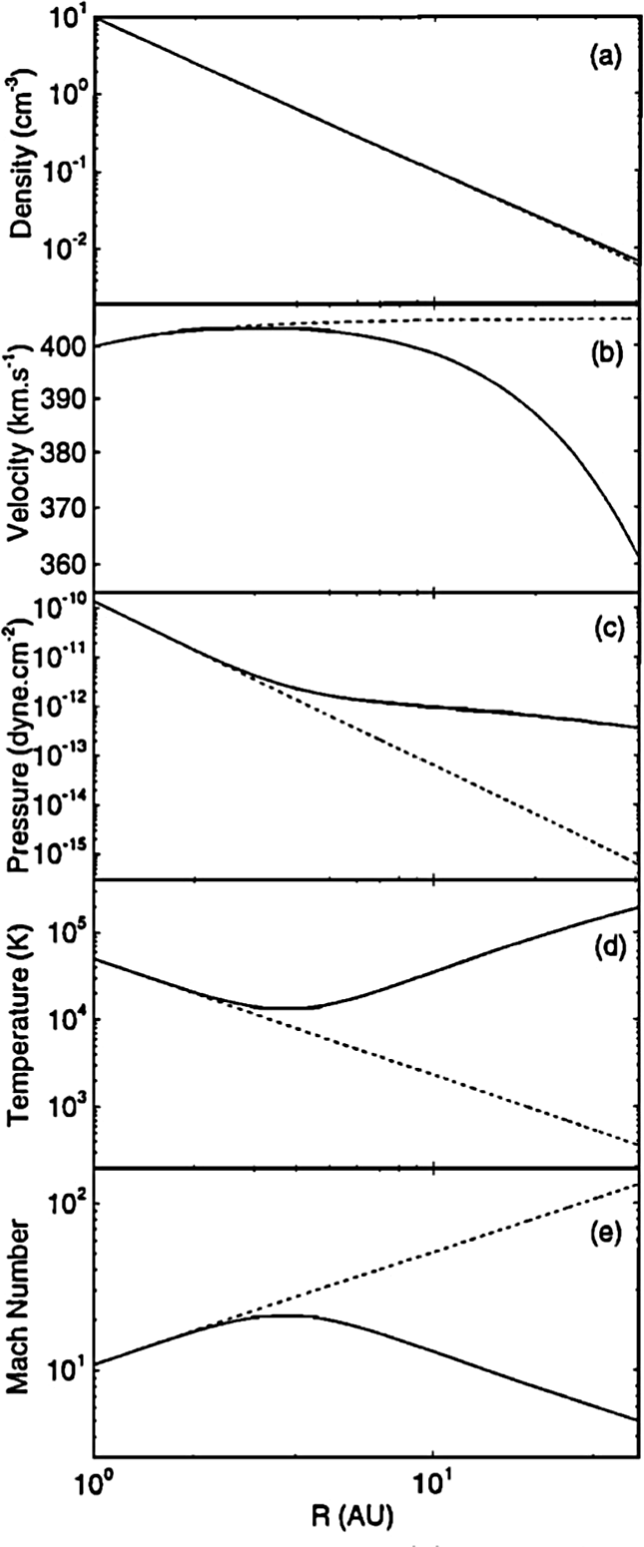
Steady state plots of (**a**) number density, (**b**) radial flow velocity, (**c**) gas pressure, (**d**) temperature, and (**e**) Mach number as a function of heliocentric distance $R$. Dashed lines correspond to an adiabatic model, and solid lines correspond to a pickup ion mediated model of the heliosphere (Zank and Pauls 1997)

Plotted in Fig. 5 is the steady-state solution to the model equations (21)–(23) subject to the assumptions that $N (r) =N_{H\infty} \exp \left [ -\lambda /r \right ]$, $T_{H} = T_{H\infty} = 10^{4}~\text{K}$, and $V = V_{H\infty} = 20\text{ km}/\text{s}$. Here $\lambda = 4~\text{AU}$ defines the ionization cavity length scale, $\nu _{ph} = 0$, and the adiabatic index $\gamma = 5/3$. The dashed lines in Fig. 5 depict a solution for which PUIs are absent, i.e., the momentum and energy source terms are set to zero and the solar wind is purely adiabatic. The solid lines depict the steady-state solar wind when PUIs are included explicitly and, while the density continues to fall off essentially as $r^{-2}$ with increasing heliocentric distance, considerable differences in the radial profiles for pressure, temperature, Mach number and velocity are apparent. Care should be exercised in interpreting the temperature profile, however. The increase in solar wind temperature corresponds primarily to the temperature of pickup ions (which have pick up energies of $\sim 1\text{ keV}$) and not to solar wind protons (which may experience some heating via both (weak) compression and turbulent dissipation – see Zank et al. (2018a) and references therein). Nonetheless, the presence of a hot pickup ion population, whose internal energy dominates that of the solar wind and which is coupled to the solar wind by scattering off *in situ* and self-generated turbulence, can be expected to affect the dynamics of local processes in the outer heliosphere considerably. Examples include the role of pickup ion pressure in the outer heliosphere (Burlaga et al. 1994), pickup ion acceleration at interplanetary shocks (Gloeckler et al. 1994a; Zank et al. 1996b; Lee et al. 1996), and the propagation of shocks (Zank and Pauls 1997). One should note the importance of PUIs for understanding the solar wind in the outer heliosphere. The presence of pickup ions in the outer heliosphere allows one to adopt a primarily hydrodynamic description rather than a model in which the outer heliosphere is dominated by the interplanetary magnetic field. For the latter case, the plasma beta (ratio of gas to magnetic field pressure) at 60 AU $\beta (60~{\text{AU}}) \simeq 0.01$. By contrast, the contribution by pickup ions yields a corresponding value of $\beta (60~{\text{AU}}) \simeq 3$ (Zank et al. 1995, 1996b).

An important extension to the one-fluid solar wind model discussed above was presented by Isenberg (1986). One-fluid solar wind models assume essentially that wave-particle interactions proceed sufficiently quickly that pickup ions are rapidly assimilated into the solar wind, becoming indistinguishable from solar wind protons. As illustrated in Fig. 5, a substantial increase in the solar wind temperature with increasing heliocentric distance is then predicted. Such a predicted temperature increase is, of course, not observed in the outer heliosphere (Gazis et al. 1994; Richardson et al. 1995) (although much more modest heating is observed beyond $\sim20~\text{AU}$, and this is now ascribed to heating via the dissipation of turbulence excited in part by the creation of PUIs and the subsequent driving of turbulence in the distant heliosphere Williams et al. 1995; Zank et al. 1996a; Matthaeus et al. 1999; Zank et al. 2018b). As observed by Vasyliunas and Siscoe (1976) and Holzer (1979), pickup ions are not assimilated into the solar wind completely. Instead, the pickup ion driven waves may isotropize and so stabilize the pickup ion distribution (and perhaps provide some residual heating of the solar wind protons). Thus, wave-particle interactions can be expected to produce two co-moving thermal proton populations. Further assimilation of the pickup ions into the solar wind distribution proceeds via Coulomb collisions. The various interaction time scales accessible to the pickup ion and solar wind proton populations were analyzed by Isenberg (1986) and Zank et al. (2014), confirming that equilibration of PUIs and thermal solar wind plasma cannot occur in the supersonic solar wind. Modern models (Isenberg 1986; Zank et al. 2018b) incorporate distinct description of PUIs and thermal plasma, including the role of turbulence and its dissipation and heating of the thermal plasma (Zank et al. 2018b), and are discussed elsewhere in this journal.

### Global Models Circa 1990

The dynamical or ram pressure ($\rho u^{2}$) and thermal pressure $p$ of the solar wind decrease with increasing heliocentric distance and must reach a value that eventually balances the pressure exerted by the LISM. The relaxation towards pressure equilibrium between the solar and interstellar plasmas is characterized by (i) a transition of the supersonic solar wind flow to a subsonic state, and (ii) a divergence of the interstellar flow about the heliospheric obstacle. The transition of the supersonic solar wind is accomplished by means of a shock transition, the heliospheric termination shock (denoted by HTS or sometimes TS). Voyager 1 crossed the HTS at 94 AU on 16 December 2004 at heliographic coordinates of ($34.3^{ \circ}$, $173^{\circ}$), i.e., in the northern hemisphere (Stone et al. 2005; Burlaga et al. 2005) and Voyager 2 crossed at 84 AU on 30 August 2008 at ($-27.5^{\circ}$, $216^{\circ}$), i.e., in the southern hemisphere (Stone et al. 2008; Burlaga et al. 2008). The divergence of the LISM flow about the heliosphere may be accomplished either adiabatically if the relative motion between the sun and the LISM is subsonic, or by means of a bow shock in the case of supersonic relative motion.

Although one can estimate the location of the HTS and the heliopause (HP), the discontinuity separating solar wind material from the interstellar plasma (a contact discontinuity in the case of gas dynamics), using simple pressure balance arguments and/or a combination of *in-situ* Voyager/LECP ions and remotely sensed Cassini/INCA ENAs (e.g. (Krimigis et al. 2011) in the Voyager 1 direction (Dialynas et al. 2020) in the Voyager 2 direction), the problem of the interaction of the solar wind with the LISM is fundamentally multi-dimensional. Thus, the main advances in our understanding of global heliospheric structure since the pioneering work of Davis (1955), Parker (1958, 1961, 1963), Axford et al. (1963), Baranov et al. (1971) have been more recent and based largely on computer simulations. The initial simulations were based on purely one-fluid gas dynamic models and only since the mid-1990s has the inclusion of neutral interstellar Hydrogen been considered at various levels of self-consistency (Baranov and Malama 1993; Pauls et al. 1995; Zank et al. 1996d). We survey briefly the analytic gas dynamic global models and the results from gas dynamic simulations, and then discuss an analytic model that incorporated the interplanetary and interstellar magnetic fields.

The quasi-analytic models of the heliosphere developed primarily by Parker (1961, 1963) and Baranov et al. (1971) largely shaped our thinking about the structure of the heliosphere until the mid-1990s. In the absence of magnetic fields, the interaction of the solar wind with the LISM is governed by the usual gas dynamic equations,

31 $$\begin{aligned} \frac{\partial \rho}{\partial t} + \nabla \cdot \rho {\mathbf{u}} &= 0; \end{aligned}$$

32 $$\begin{aligned} \frac{\partial}{ \partial t} \left ( \rho {\mathbf{u}} \right ) + \nabla \cdot \left ( \rho {\mathbf{u}} {\mathbf{u}} \right ) + \nabla p &= 0; \end{aligned}$$

33 $$\begin{aligned} \frac{\partial}{ \partial t} \left ( \frac{1}{2} \rho u^{2} + \frac{p}{\gamma - 1}\right ) + \nabla \cdot \left ( \frac{1}{2} \rho u^{2} {\mathbf{u}} + \frac{\gamma}{\gamma - 1} {\mathbf{u}} p \right ) &= 0, \end{aligned}$$

where, as before, $\rho $, ${\mathbf{u}}$, and $p$ denote plasma density, velocity, and total pressure (thermal ions and electrons). The adiabatic index $\gamma = 5/3$. As we discuss below, source terms should be included in equations (31)–(33) that couple the plasma and the neutral H distribution and, in principle, one needs to compute the evolution of both distributions simultaneously. Effects such as heating due to the dissipation of turbulence are absent. For a steady, radially symmetric solar wind interacting with a static, unmagnetized interstellar gas (Davis 1955; Parker 1958, 1961, 1963), the deceleration of the expanding supersonic solar wind must be accomplished by a strong shock (at least within the framework of a gas dynamic solar wind in the absence of pickup ions) for which the Rankine-Hugoniot conditions normal to the shock are

34 $$ \frac{u_{2}}{u_{1}} \simeq \frac{\gamma - 1}{\gamma + 1} ; \quad \frac{\rho _{2}}{\rho _{1}} \simeq \frac{\gamma + 1}{\gamma - 1} ; \quad p_{2} \simeq \frac{2}{\gamma + 1} \rho _{1} u_{1}^{2} . $$

The subscript 1(2) denotes upstream (downstream) states i.e., 1 refers to the supersonic solar wind. Since the downstream Mach number is small, we may assume that the flow there is incompressible ($\rho _{2}$ constant). Along each streamline in the downstream region, the Bernoulli equation is valid i.e.,

35 $$ p + \frac{1}{2} \rho u^{2} = p_{2} + \frac{1}{2} \rho _{2} u_{2}^{2}. $$

Then, since $p \rightarrow p_{\infty}$, the LISM pressure, and $u \rightarrow 0$ at the stagnation point, $p_{2} + \rho _{2} u_{2}^{2} / 2 = p_{\infty}$. Making the further assumption that the solar wind speed $u_{1} = u_{0}$ is constant, where the subscript 0 denotes evaluation at 1 AU, implies that

36 $$ \rho r^{2} = \rho _{0} r_{0}^{2} = \rho _{t} R_{t}^{2} . $$

Here $R_{t}$ denotes the location of the termination shock. It follows immediately from (34)–(36) that the HTS is located at

37 $$ \frac{R_{t}}{r_{0}} = \left [ \frac{\gamma + 3}{2(\gamma + 1)} \frac{\rho _{0}u_{0}^{2}}{p_{\infty} } \right ]^{1/2} . $$

Behind the HTS, where $\rho \simeq const.$ by assumption, the steady, spherically symmetric continuity equation (31) yields $ur^{2} = u_{2}R_{t}^{2}$, from which one obtains

38 $$ u = \frac{\gamma - 1}{\gamma + 1} u_{0} \left ( \frac{R_{t}}{r} \right )^{2} , $$

showing that the heliosphere expands slowly outward if bounded by a static interstellar plasma. For typical solar wind and LISM parameters ($u_{0} = 400~\text{km}/\text{s}$, $n_{0} = 5~\text{cm}^{-3}$, $p_{\infty} = 10^{-13}~\text{dyn}\,\text{cm}^{-2}$), $R_{t}\simeq 350~\text{AU}$.

The relative motion of the sun with respect to the interstellar plasma changes matters dramatically. Following Parker (1961, 1963), suppose that the ram pressure of the LISM is much less than the thermal pressure i.e., that $\rho _{\infty} u_{\infty}^{2} \ll p_{\infty}$, which implies that $M_{\infty} \ll 1$. To determine the flow pattern of the subsonic solar wind, we follow the presentation of Suess and Nerney (1990) (see also Khabibrakhmanov and Summers 1996). Suppose that the supersonic flow terminates at a spherical termination shock located at the radial distance $R_{t}$. Since flow downstream of the HTS is approximately incompressible, $\nabla \cdot {\mathbf{u}}= 0$, or in terms of the velocity potential ${\mathbf{u}} = \nabla \phi $,

39 $$ \nabla ^{2} \phi = 0 . $$

The flow pattern can then be determined from the general solution of Laplace’s equation (39). The velocity potential can be expressed as a linear combination of the potential of three different sources,

$$ \phi _{1} = u_{\infty} r \cos \theta , \quad \phi _{2} = \frac{m \cos \theta }{4 \pi r^{2}} , \quad \phi _{3} = \frac{Q}{4\pi r} , $$

where, respectively, they are (1) the potential of the steady flow along the $z$-axis, (2) the potential of the dipole, with moment ${\mathbf{m}}$, aligned along the $z$-axis at the center of the sun, and (3) the potential of the source of strength $Q$ located at the origin. The flow is obviously axisymmetric. The velocity potential is (Suess and Nerney 1990)

40 $$ \phi = u_{\infty} R_{t} \cos \theta \left [ \frac{1}{2} \left ( \frac{R_{t}}{r} \right )^{2} + \frac{r}{R_{t}} \right ] + \frac{u_{t} R_{t}^{2}}{r} . $$

Since $u_{\infty} = -3/2 u_{\infty} \sin \theta $ at the HTS (i.e., an azimuthal component of ${\mathbf{u}}$), the assumption of a spherically symmetric HTS is not in fact valid and one is obliged then to compute the HTS geometry self-consistently with the flow pattern. The solution (40) is therefore valid only for small $R_{t}$ and the term $(R_{t} /r)^{2}$ represents a correction to Parker’s (Parker 1961, 1963) original point source solution ($R_{t}= 0$).

The stream function $\Psi $ is given by

41 $$\begin{aligned} \Psi = - \frac{u_{\infty} R_{t}^{3}}{2r} \left [ 1 - \left ( \frac{r}{R_{t}} \right )^{4} \right ] \sin ^{2} \theta - u_{t} R_{t}^{2} (\cos \theta - 1 ) , \end{aligned}$$

in spherical coordinates (Nerney et al. 1993; Khabibrakhmanov and Summers 1996). The stagnation point $R_{H}$ of the interstellar wind is found on the stagnation line $\cos \theta = 1$, $u_{r} = 0$, i.e.,

42 $$ \frac{u_{\infty} }{u_{t}} \left [ \left ( \frac{R_{t}}{R_{H}} \right )^{3} - 1 \right ] + \left ( \frac{R_{t}}{R_{H}} \right )^{2} = 0, $$

which generalizes the Parker (1961) solution $\left ( R_{t} / R_{H} \right )^{2}= u_{\infty} / u_{t}$. The shape of the heliopause is determined by the null surface of the streamline function $\Psi =0$,

43 $$ \left ( u_{\infty} /u_{t} \right ) \left [ 1 - \left ( \frac{r}{R_{t}} \right )^{3} \right ] \sin ^{2} \theta - 2 \frac{r}{R_{t}} \left ( \cos \theta - 1 \right ) = 0. $$

**Fig. 6:**
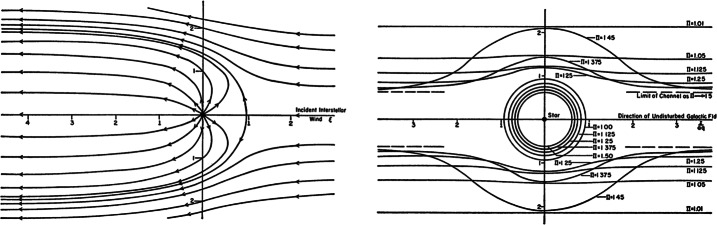
Left: From the original paper by Parker (1961), illustrating the streamlines of the subsonic, nearly incompressible, hydrodynamic flow of a stellar wind beyond the shock transition for an incident gas dynamical subsonic interstellar wind. Right: The shock transition as shown by the concentric circles, and the outer boundary of a stellar-wind region in the presence of a large-scale interstellar magnetic field, for various values of the stagnation pressure at infinity (Parker 1961)

**Fig. 7:**
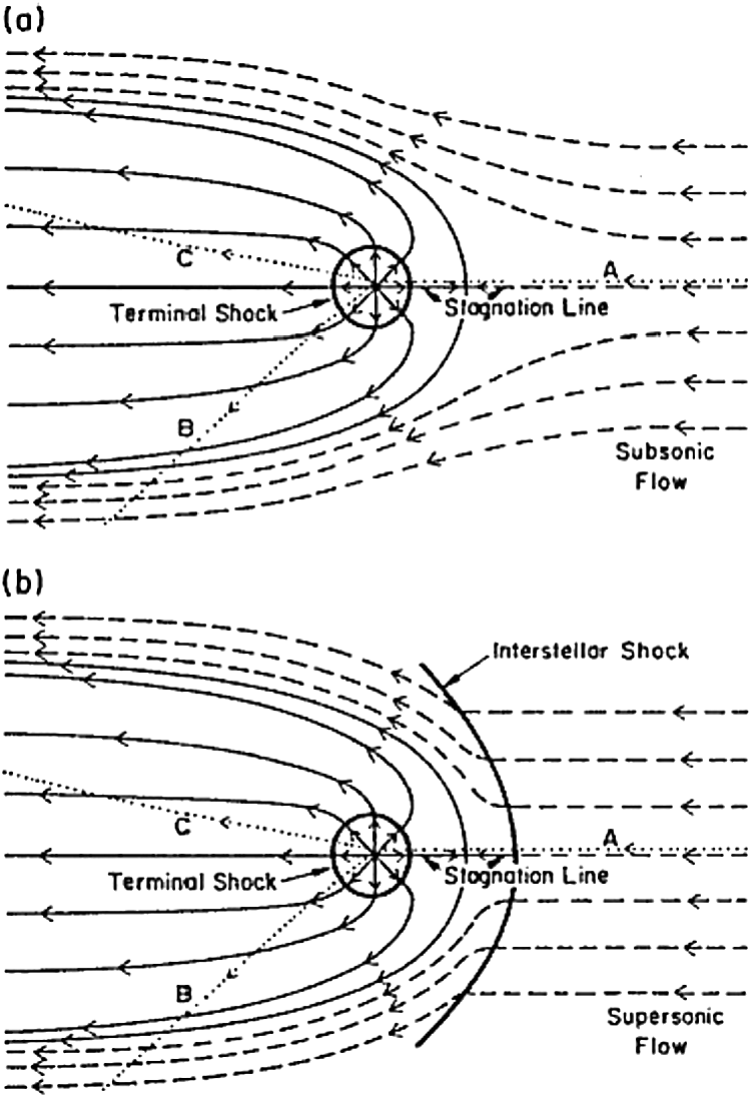
Schematic representation of streamline plots for the irrotational flow solutions of the interaction of the solar wind flow with (**a**) a subsonic interstellar medium and (**b**) a supersonic LISM. The solid curves denote solar wind plasma flow and the dashed lines LISM flow. The dotted curves are trajectories of an interstellar hydrogen atom that is subjected to either a net attractive force (AB) or a net repulsive force (AC). The termination shock, heliopause, stagnation point, heliosheath and heliotail are marked. The flows are symmetric about the stagnation axis. After Suess and Nerney (1990) and Holzer (1989)

In the limit that $r \rightarrow \infty $, the transverse dimension of the distant heliotail is

$$ R_{t} \sqrt{ 2 u_{t} / u_{\infty} } , $$

indicating that the solar wind in the heliotail is confined to a circular cylinder. Figures 6 and 7 (top) show the analytic global structure for the one-shock model. The original figures from the classic paper by Parker (1961) are provided for historical context, illustrating how his basic ideas influenced our ideas of large-scale heliospheric structure for decades. The possibility of a supersonic solar wind, discussed below, was the first important conceptual change to Parker’s original formulation, but the major paradigm shift occurred with the incorporation of interstellar neutral H and its charge-exchange coupling to plasma.

The possibility that the solar wind interacts with a supersonic interstellar wind was considered by Baranov et al. (1971). In this case, two shocks are present – a bow shock through which the interstellar flow is decelerated and diverted about the heliospheric obstacle, and a solar wind termination shock. A contact discontinuity, called the heliopause (HP), separates the heated, compressed, subsonic solar wind and the shocked LISM flows. The region between the HTS and the HP is called the inner heliosheath.

Baranov et al. (1971) treat the subsonic region as a thin shell separating hypersonic streams (i.e., the dense shell has negligible thickness compared to the distance to the Sun). This approximation is sometimes called the “thin shell” or “Newtonian” approximation. By expressing conservation of mass and momentum for the shell, which is assumed to be described by a curve of the form $r = r( \theta )$, in directions normal and tangential to the layer, one can derive a 3rd-order ordinary differential equation (Baranov et al. 1976, 1971) (see also Ratkiewicz 1992)

44 $$ r r^{\prime \prime} = \frac{F_{1} - F_{2}^{\prime} /F_{3}}{F_{2}} F_{3}^{2} + 2 rr^{\prime} + 3 r^{\prime} r^{\prime \prime} , $$

describing the shape of the discontinuity. Here $F_{1,2,3} = F_{1,2,3} (r, \theta )$. Equation (44) was solved numerically using the boundary conditions

$$ r (\theta = 0 ) = R_{B} , \quad r^{\prime} (\theta = 0) = 0 , $$

where $R_{B}$ is the heliocentric distance to the shell along the axis of symmetry. $R_{B}$ is determined from

45 $$ \rho _{1} u_{1}^{2} = \rho _{\infty} u_{\infty}^{2} , \qquad \rho _{1} u_{1} R_{B}^{2} = \rho _{0}u_{1} r_{0}^{2} = const., $$

where $u_{1}$ is the solar wind speed and the subscript 0 denotes evaluation at 1 AU. From (45), the shell length scale is given by

46 $$ R_{H} /r_{0} = \sqrt{ \frac{\rho _{0} u_{1}^{2}}{\rho _{\infty} u_{\infty} }}. $$

The third boundary condition is $r^{\prime \prime} = 2R_{B} / 5$ (Baranov et al. 1971). Unfortunately, as we now know from both simulations and direct measurements of the width of the inner heliosheath by Voyager 1 & 2, the Newtonian or thin layer approximation is rather poor and the distance between the bow shock and the HTS is comparable to the distance from the Sun. Nonetheless, the thin layer approximation has been used by a variety of authors to investigate different aspects of the solar wind-LISM interaction, including the effect of interstellar magnetic fields on global heliospheric structure.

The analytic models, while far from satisfactory, do illustrate at least three basic results of importance for a solar wind interacting with an interstellar wind. The first is that an extended tail of subsonic solar wind should form – now called the heliotail. Secondly, the supersonic region of the heliosphere should be asymmetric. Thirdly, if we neglect the deceleration of the solar wind by resonant charge exchange effects, the minimum radius to the solar wind shock transition can be calculated from equation (37) by assuming a value for the LISM pressure. The LISM pressure term can include the thermal gas, cosmic rays, the interstellar ram pressure, the magnetic field pressure, dust, and MHD turbulence, and may be expressed as

47 $$ p_{\infty} = \rho u^{2} + p_{th} + \alpha B^{2} / 2 \mu + p_{CR} + p_{dust} + p(\delta B^{2} ) , $$

where the terms are all evaluated in the LISM and the factor $\alpha $ attempts to include the effects of magnetic field obliquity. The analytic models are represented schematically in Fig. 7 for both the one-shock and two-shock cases.

To properly understand the global structure of the heliosphere requires the use of numerical simulations. Ideally, these simulations would include self-consistently the interaction of the solar wind plasma, interstellar plasma, neutrals of both interstellar and heliospheric origin, magnetic fields, etc., all within the framework of a time-dependent, multi-dimensional, highly resolved code. A modest version of this program was beginning to be implemented by the late 1980s and early to mid-1990s. The assumption of axial symmetry along the direction of the LISM flow coupled to a spherically symmetric expanding solar wind allows one to reduce the gasdynamic equations (31)–(33) (without the source terms) to a 2D model. Such a reduced model has been investigated by several groups (Baranov et al. 1979; Matsuda et al. 1989; Baranov and Malama 1993; Steinolfson et al. 1994; Steinolfson 1994; Karmesin et al. 1995; Pauls et al. 1995; Wang and Belcher 1998) and the purely gas dynamic simulations are well understood. We describe two representative 2D gas dynamical examples for the parameters tabulated in Table 1. The LISM temperature is chosen so that it is either supersonic ($T = 8{,}000~\text{K}$) or subsonic ($T = 80{,}000~\text{K}$). Clearly, the latter temperature is an effective temperature, reflecting the possible pressure contribution from e.g., low energy cosmic rays or the magnetic field). An alternative approach is to simply reduce the LISM flow velocity, e.g., Steinolfson et al. (1994).

**Table 1:** Solar wind parameters at 1 AU and LISM parameters at infinity used for the simulations illustrated in Fig. 8

	Solar wind (1 AU)	LISM
*n* (cm^−3^)	5.0	0.1
*u* (km/s)	400	−26
*T* (K)	10^5^	8,000/80,000
*M*	7.6	1.75/0.9

**Fig. 8:**
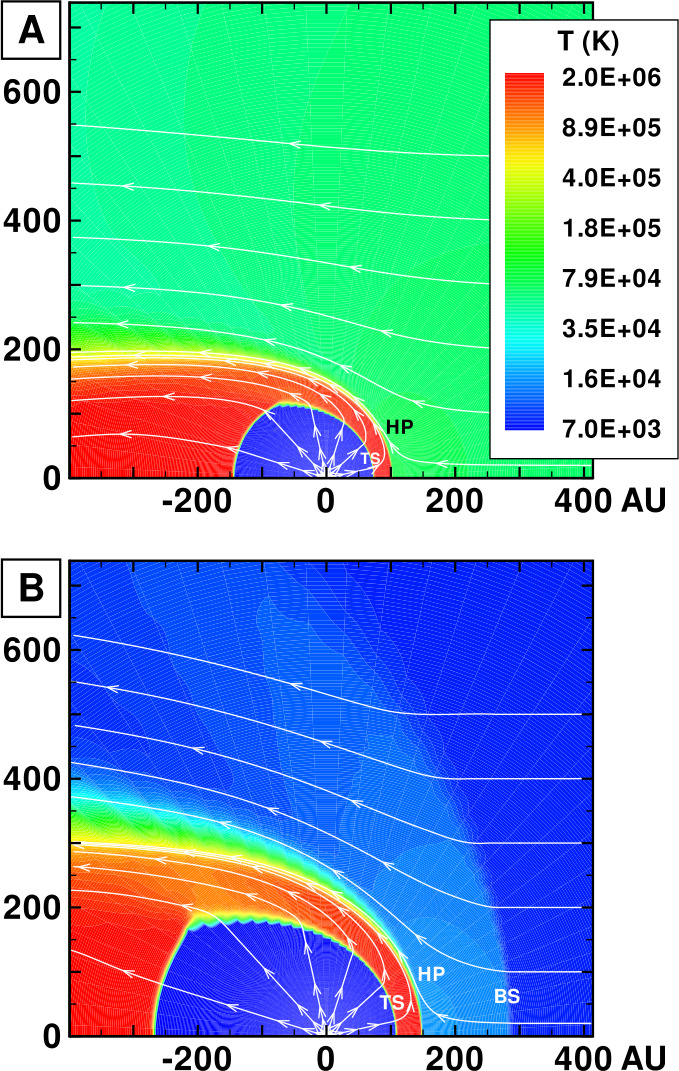
(**A**) Contour plot of the Log[Temperature] for a 2D gas dynamic 1-shock model. The heliospheric termination shock (HTS or TS) and the heliopause (HP) are shown. (**B**) As with (A) except now for a 2-shock model. A bow shock (BS) is now present (Zank 1999a). Revised figures courtesy of H.-R. Müller

**Fig. 9:**
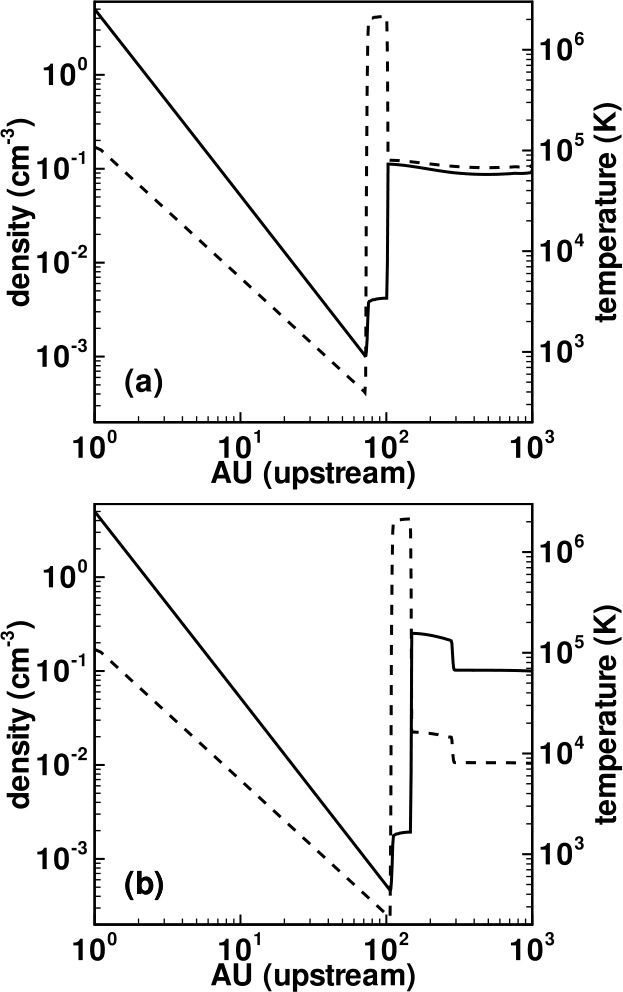
One-dimensional cuts along the stagnation axis of the plasma-only 2D simulations for (**a**) the one-shock model, and (**b**) the two-shock model. The solid line shows the plasma density and the dashed line the plasma temperature. The high LISM plasma temperature for the one-shock model is an effective temperature, reflecting the contribution of cosmic rays, for example. The HTS, HP and BS are all clearly visible (Zank 1999a). Revised figures courtesy of H.-R. Müller

Illustrated in Fig. 8A is the temperature distribution (color) of the plasma at steady-state for an assumed subsonic interstellar medium. The location of the heliopause (HP) and termination shock (HTS) are labelled. Also shown in Fig. 9a are 1D profiles of the plasma variables $\rho $ and $T$ in the nose (i.e., along the stagnation line) direction. Figure 8 shows that the incoming subsonic LISM flow is decelerated and diverted far upstream of the heliospheric obstacle and some associated adiabatic compression and heating of the LISM plasma occurs ahead of the heliopause. Nonetheless, the flow lines are qualitatively consistent with the analytic streamlines derived in Parker (1961, 1963), Suess and Nerney (1990). The interstellar and solar wind plasma meet at the heliopause where they flow at different tangential speeds. The termination shock is located at $\sim 70~\text{AU}$ in the upwind direction and is very strong (compression ratio of 4). Considerable heating of the plasma occurs and downstream (inner heliosheath) temperatures are typically $\sim 10^{6}~\text{K}$. Although the heliosheath, the region between the HTS and HP, expands like a de Laval nozzle, the shocked solar wind does not expand sufficiently rapidly to become supersonic, remaining subsonic throughout the heliosheath. The 2D structure of the HTS resembles a slightly elongated sphere. The downstream HTS is located at $\sim 78~\text{AU}$ which is scarcely further than the distance in the upstream direction. The shocked solar wind in the heliotail does not cool with increasing distance from the HTS and is effectively cylindrical, as expected from the analytic models.

At the heliopause (HP), a contact discontinuity in these gas dynamic models, the density increases abruptly and dramatically from the shocked solar wind value to one almost equal to the undisturbed LISM density, as illustrated in Fig. 9A. A corresponding abrupt decrease in plasma temperature occurs here as well. The heliosheath is approximately 50 AU wide in the upstream direction.

Suppose now that the LISM flow is supersonic. In this case, as discussed above, a bow shock (BS) is necessary to divert the approaching LISM flow about the heliosphere. Figure 8B shows the plasma temperature at steady-state when charge exchange with neutrals is neglected. The positions of the HTS, HP and BS are indicated on the plot. For this purely gas dynamic case, the HTS is axisymmetric and bullet shaped (Baranov and Malama 1993; Steinolfson et al. 1994; Pauls et al. 1995; Steinolfson and Gurnett 1995; Wang and Belcher 1998). The HTS has a compression ratio of $r = 4$ independent of polar angle, while the BS on the other hand is a weak shock ($r=1.7$ at $\theta =0^{\circ}$). The supersonic solar wind flow velocity is radial and constant, resulting in an adiabatic expansion of the plasma ($\rho \propto r^{-2}$, $T\propto r^{-4/3}$) up to the HTS, where the density, pressure and velocity of the plasma jump discontinuously to a subsonic flow that is deflected towards the heliotail ($\theta =180^{\circ}$). The LISM and solar wind plasmas meet at the HP where the interstellar and shocked solar wind pressures are balanced, while the density of the plasma jumps discontinuously. The width of the heliosheath (distance between the HTS and HP) at the nose ($\theta =0^{\circ}$) is $\sim 50~\text{AU}$ and the upstream HTS is located at $\sim 120~\text{AU}$. The heliocentric distance of the BS along the stagnation axis is $\sim 330~\text{AU}$.

Consider the supersonic solar wind flow at $\theta = 0^{\circ}$ (the nose of the heliosphere), as shown in Fig. 8B. The plasma is shocked at the HTS, and the subsonic heated plasma flows from the heliosheath to the tail region. Moving about the flanks of the HTS, the flow accelerates to a supersonic state as shown by the presence of a sonic line (Mach number $M = 1.0$) in the heliosheath (Pauls et al. 1995). Since the supersonic heliosheath flow must eventually accommodate to the subsonic flow in the heliotail, a shock wave attached to the HTS is necessary, together with an additional contact discontinuity. As is evident from Fig. 8B, the supersonic flow shocks once again at the reflected shock and the re-shocked material meets the heliotail flow at a contact discontinuity. 1D cuts of the density and temperature along the stagnation axis are illustrated in Fig. 7b.

The 2D models discussed above assumed the solar wind to be independent of heliolatitude. However, as revealed by the Ulysses spacecraft during its pass over the southern pole of the Sun (Phillips et al. 1995), the structure of the solar wind is clearly three-dimensional during solar minimum periods. The interplanetary magnetic field (IMF) also renders heliospheric structure intrinsically 3D. Pauls and Zank (1996) neglected the IMF to provide the first detailed 3D numerical model of the solar wind interaction with the LISM, focussing on the solar minimum and maximum conditions. Measurements of the solar wind speed during the southern polar pass indicate that the solar wind speed increases from $\sim400~\text{km}\,\text{s}^{-1}$ in the ecliptic plane to $\sim700~\text{km}\,\text{s}^{-1}$ over the Sun’s pole, while the proton number density decreases from $\sim 8$ to $\sim3~\text{cm}^{-3}$ in going from ecliptic plane to the Sun’s pole. The observed proton temperature also increases from $\sim50{,}000~\text{K}$ to $\sim200{,}000~\text{K}$ from ecliptic to pole (Phillips et al. 1995). The heliolatitudinal variation results in an increase, by a factor of about 1.5, in the momentum flux in going from the ecliptic plane to the solar pole. These observations correspond to a solar wind during solar minimum comprising two components, the first being a steady, long-lived, hot, low-density, high-speed wind emanating from two large polar coronal holes, one in each hemisphere and extending down to about $35^{\circ}$ heliolatitude, and bounding a cool, sluggish, high-density, somewhat turbulent solar wind.

**Fig. 10:**
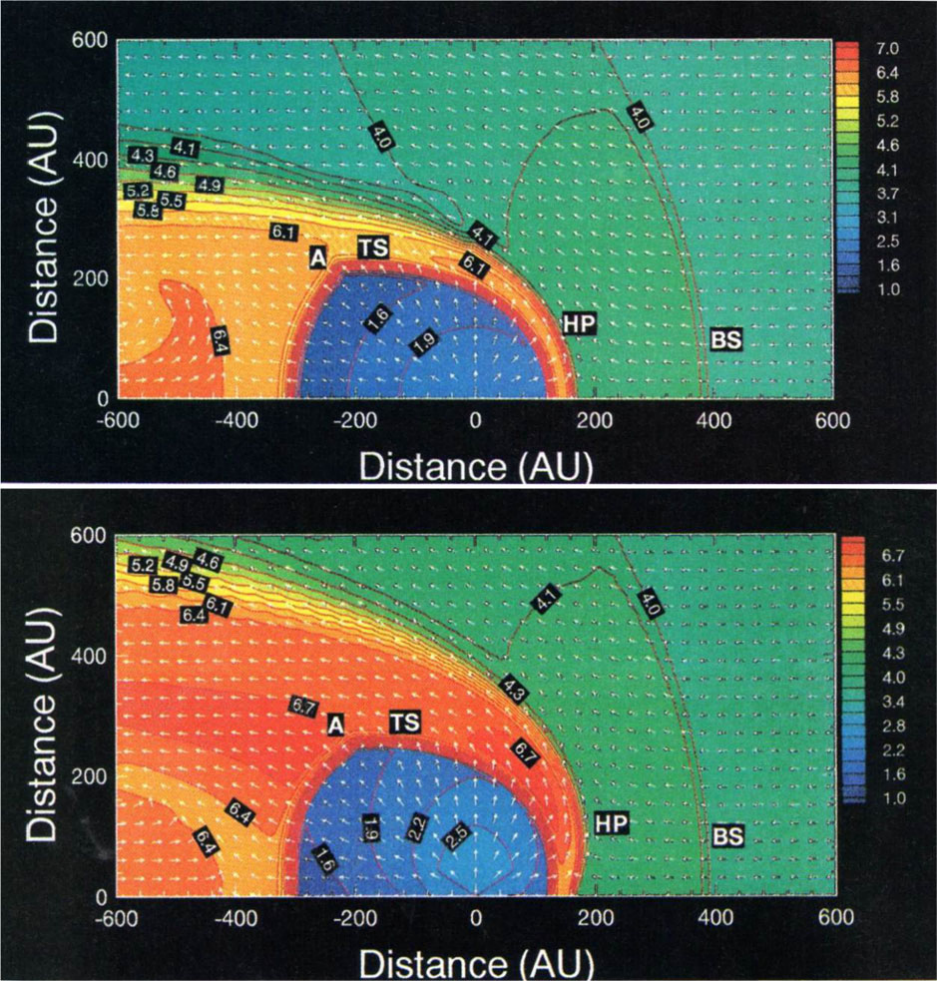
(Top) Log[Temperature] (contour and color) and normalized flow vectors in the ecliptic plane for a two-shock anisotropic solar wind. The position of the triple point is identified by A, the termination shock by TS, the heliopause by HP, and the bow shock by BS. (Bottom) As in the left plate except now for the polar plane (Pauls and Zank 1996)

Pauls and Zank (1996) extended their 2D gas dynamic models to 3D to include heliolatitudinal variation in the solar wind. Barnes (1998) investigated analytically the shape of the termination shock when latitudinal variation of the solar wind ram pressure is included. On using initial data that correspond to the Ulysses heliographic plasma observations, the 3D structure of the large-scale heliosphere is illustrated in Fig. 10 (Pauls and Zank 1996). Two planar cuts through the three-dimensional heliosphere for a supersonic LISM show the $\log T$ and normalized flow vectors in a cut through the ecliptic plane (Fig. 10 (top)), while the bottom plate shows these values in the polar plane. Comparing these two plots, one observes that the HTS is bullet-shaped and elongated along the poles of the Sun. The elongation results from the increased solar wind ram pressure with heliolatitude, hence the increase in distance to the HTS and consequently the HP over the poles of the Sun. Since the solar wind ram pressure increases as a function of heliolatitude, the distance to the HTS in the polar plane (shown in Fig. 10 (top)) increases more rapidly with heliolatitude than that of an isotropic solar wind. This leads to a slightly higher pressure at the stagnation point for an anisotropic solar wind than an isotropic solar wind. The higher pressure forces the bow shock further out (350 AU for an isotropic solar wind compared to 380 AU for an anisotropic solar wind). Another consequence of the ram pressure increase with heliolatitude is the rapid increase in distance to the HP with latitude in the region of the nose, as can be seen from Fig. 10. Although not shown, a cut through the three-dimensional solution in a plane perpendicular to the stagnation line through the origin, reveals an hour glass shape for the HP, with the elongation along the solar poles.

The width of the heliosheath at the nose decreases from 53 AU in the isotropic solar wind case to 30 AU for the anisotropic solar wind (Pauls and Zank 1996). The reason for the decrease is a consequence of an increase in the LISM ram pressure acting on the HP. The shocked LISM flow for the anisotropic solar wind case is forced to flow to the heliotail in the ecliptic plane rather than uniformly over the heliosphere as is the case for the isotropic solar wind. This then leads to an increase in the LISM ram pressure acting on the interstellar side of the HP. The same argument holds for the heliosheath flow, but to a lesser extent. This enhanced shocked LISM ram pressure forces the HP closer to the Sun.

### Role of the Magnetic Field in Global Models

To model the interaction of the solar wind with a partially ionized LISM, the following 3D set of MHD equations is typically solved,

48 $$\begin{aligned} \frac{\partial \rho}{ \partial t} + \nabla \cdot \rho {\mathbf{u}} &= Q_{ \rho} ; \end{aligned}$$

49 $$\begin{aligned} \rho \frac{\partial {\mathbf{u}}}{ \partial t} + \rho {\mathbf{u}} \cdot \nabla {\mathbf{u}} + (\gamma - 1) \nabla e + \left ( \nabla \times {\mathbf{B}} \right )\times {\mathbf{B}} &= {\mathbf{Q}}_{m} ; \end{aligned}$$

50 $$\begin{aligned} \frac{\partial}{ \partial t} \left ( \frac{1}{2} \rho u^{2} + e + \frac{B^{2}}{8\pi} \right ) + \nabla \cdot \left [ \left ( \frac{1}{2} \rho u^{2} + \gamma e \right ){\mathbf{u}} + \frac{1}{4\pi} { \mathbf{B}} \times ({\mathbf{u}} \times {\mathbf{B}} ) \right ] &= Q_{e}; \end{aligned}$$

51 $$\begin{aligned} \frac{\partial {\mathbf{B}} }{\partial t} - \nabla \times \left ( {\mathbf{u}} \times{\mathbf{B}} \right ) &= 0; \end{aligned}$$

52 $$\begin{aligned} \nabla \cdot {\mathbf{B}} &= 0 , \end{aligned}$$

$\rho = m_{p} n$, together with the equation of state $e = \alpha n k_{B} T/ (\gamma - 1) =p/(\gamma - 1)$. Here the choice of $\alpha = 1$ or 2 (or greater) corresponds to either the neutral or plasma population. The remaining variables have their usual definitions and the source terms $Q_{\rho}$, ${\mathbf{Q}}_{m}$, and $Q_{e}$ serve to couple the neutral hydrogen and proton populations. Subject even to the assumption of an isotropic solar wind, the problem (48)–(52) is inherently 3D thanks to the solar magnetic field and the current sheet.

In the case that neutral H is included through the source terms in Eqs. (48)–(52), the model equations would include pickup ions through the pressure term $p$, but the PUIs are coupled tightly to the background thermal plasma through the explicit assumption that the PUI heat flux is zero. Pick-up ions are created in the solar wind through charge-exchange of LISM neutrals with solar wind protons, but they do not thermalize with the background solar wind plasma Isenberg (1986), Zank et al. (2014) and are not therefore equilibrated with the solar wind.

In this subsection, we explicitly neglect the inclusion of neutral H for the present and consider the important role of both the interplanetary and interstellar magnetic fields in determining the global structure of the heliosphere and its boundaries. The interstellar magnetic field (ISMF) strength and orientation affect the shape and position of the heliopause relative to the Sun and interstellar plasma velocity vector, originally discussed by Fahr et al. (1988), Baranov and Zaitsev (1995), Washimi and Tanaka (1996), Linde et al. (1998), Pogorelov and Matsuda (1998), and Ratkiewicz et al. (1998). The interplanetary magnetic field, by virtue of the current sheet, introduces a corresponding asymmetry inside the inner heliosheath that also affects the shape and position of the heliopause (Washimi and Tanaka 1996; Linde et al. 1998; Zank 1999a).

Much of the interplanetary magnetic behavior in global heliospheric models can be understood on the basis of simple kinematic models. Magnetic fields do not play a major role in the dynamics of the supersonic solar wind, their pressure contribution being much less than that of the solar wind ram pressure. However, in the presence of a solar wind decelerated by ion pickup, the magnetic field can deviate slightly from the expectations of the familiar (Parker 1963) interplanetary magnetic field. Much more interesting is the role of the magnetic field in the heliosheath, especially in the upstream region where the flow is subsonic and approximately incompressible. It was observed by Axford (1972) and Cranfill (1971) that downstream of the termination shock, the flow velocity $u \propto r^{-2}$ for a flow that is spherically symmetric, radial and incompressible ($\rho \sim \mathrm{const.}$). Hence, since

53 $$ r u B_{\phi} = \mathrm{constant}, $$

($B_{r}$ can safely be neglected), the azimuthal or transverse component increases according to

54 $$ B_{\phi} \propto r \sin \theta . $$

Evidently, the magnetic field energy density must increase downstream of the termination shock until it begins to exert an appreciable dynamical effect on the diverted shocked solar wind flow and the interstellar flow. Indeed, Korolkov and Izmodenov (2021) have further explored the dynamic effect of the azimuthal magnetic field in the heliosheath/astrosheath in the context of ideal MHD, finding that it can become so large as to change of topology of heliopause/astropause

Nerney et al. (1991, 1993, 1995) have used the heliospheric model of Suess and Nerney (1990) to compute kinematically the interplanetary magnetic field in the heliosheath. In particular, they suggested on the basis of their gas dynamic generalization of Parker’s model that the distance separating the heliopause and termination shock might be $R_{H}/R_{t} \simeq 2$, $R_{H}$ the distance to the HP, with the implication that the Axford-Cranfill effect might be negligible. To quantify the importance of the Axford-Cranfill effect, the induction and solenoidal equations (51) and (52) can be solved in the (kinematic) limit in which the force on the fluid by the magnetic field is ignored (Nerney et al. 1991). Zank (1999a) used the Pauls and Zank (1996) 3D hydrodynamical model of an isotropic solar wind interacting with the supersonic LISM to investigate the kinematic magnetic field topology under more realistic flow conditions. A related approach to investigate the kinematic magnetic field in the inner heliosheath using the model of Baranov and Malama (1993, 1996) was presented by Barsky (1999).

**Fig. 11:**
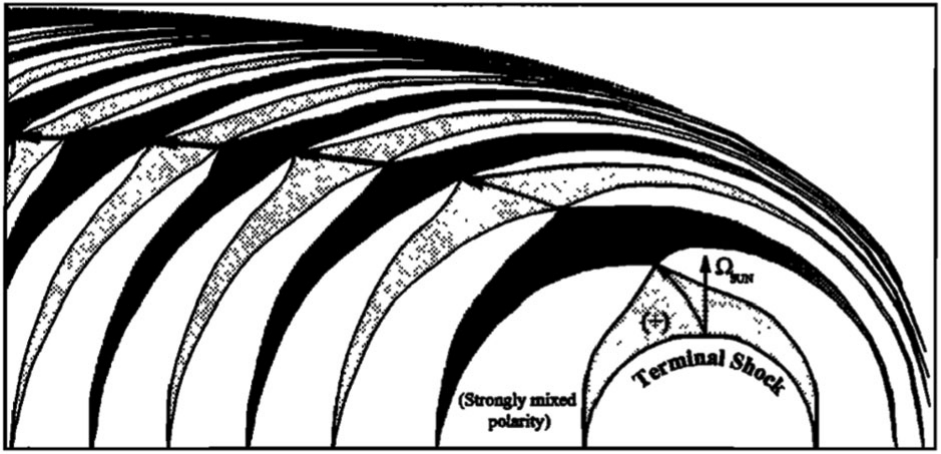
Outline of unipolar magnetic envelopes in the heliosheath in the $y-z$-plane. Each envelope is associated with one solar sunspot cycle. Between the envelopes the field is strongly mixed due to the 27-day sector pattern. The path of the single polar magnetic field line is shown, along with the reversal of the field polarity along this path every solar sunspot cycle (Nerney et al. 1995)

Before turning to simulations in which the magnetic field is included self-consistently, it should be mentioned that Nerney et al. (1995) considered the effect of the 22-year solar cycle on the heliosheath magnetic field. Since the heliosheath flow speed is subsonic for large regions of the two-shock model and throughout the one-shock model, it can take almost one solar cycle for the shocked solar wind to convect from the heliosheath into the heliotail. Thus, the changing polarity of the solar magnetic field over the solar cycle can influence the overall magnetic field structure in the heliosheath. The streamlines in the heliosheath carry an imprint of the 11-year magnetic polarity cycle, which Nerney et al. describe as “magnetic polarity envelopes” (Nerney et al. 1995). The polarity envelopes are mappings of the unipolar regions lying at either pole of the sun, illustrated in Fig. 11. During solar minimum, the polarity envelopes grow to their maximum latitudinal extent and shrink to zero at solar maximum. This leads to alternating polarities of the envelopes in the heliosheath.

Both the direction and magnitude of the LISM magnetic field were for a long time perhaps the poorest constrained external boundary conditions for the heliosphere. Although interplanetary and interstellar magnetic fields renders the problem of heliospheric structure fully three-dimensional, some efforts were made nonetheless to include magnetic fields into axisymmetric models (Fujimoto and Matsuda 1991; Baranov and Zaitsev 1995; Pogorelov and Semenov 1997). This was done by assuming that the LISM magnetic field is parallel to the interstellar flow and that the solar wind is not magnetized. As we now know, such a field orientation is not appropriate to the LISM. Early basic 3D MHD results were presented by Washimi (1993), Washimi and Tanaka (1996), Ratkiewicz et al. (1998), Pogorelov and Matsuda (1998), and Linde et al. (1998). Ratkiewicz et al. (1998) assume an unmagnetized solar wind and consider the variation of interstellar magnetic field strength and orientation. Washimi and Tanaka (1996) and Linde et al. (1998) developed fully 3D MHD simulations that include the interplanetary magnetic field. We consider first the simulations of Washimi & Tanaka since, unlike those by Linde et al., no neutral hydrogen model is included.

**Fig. 12:**
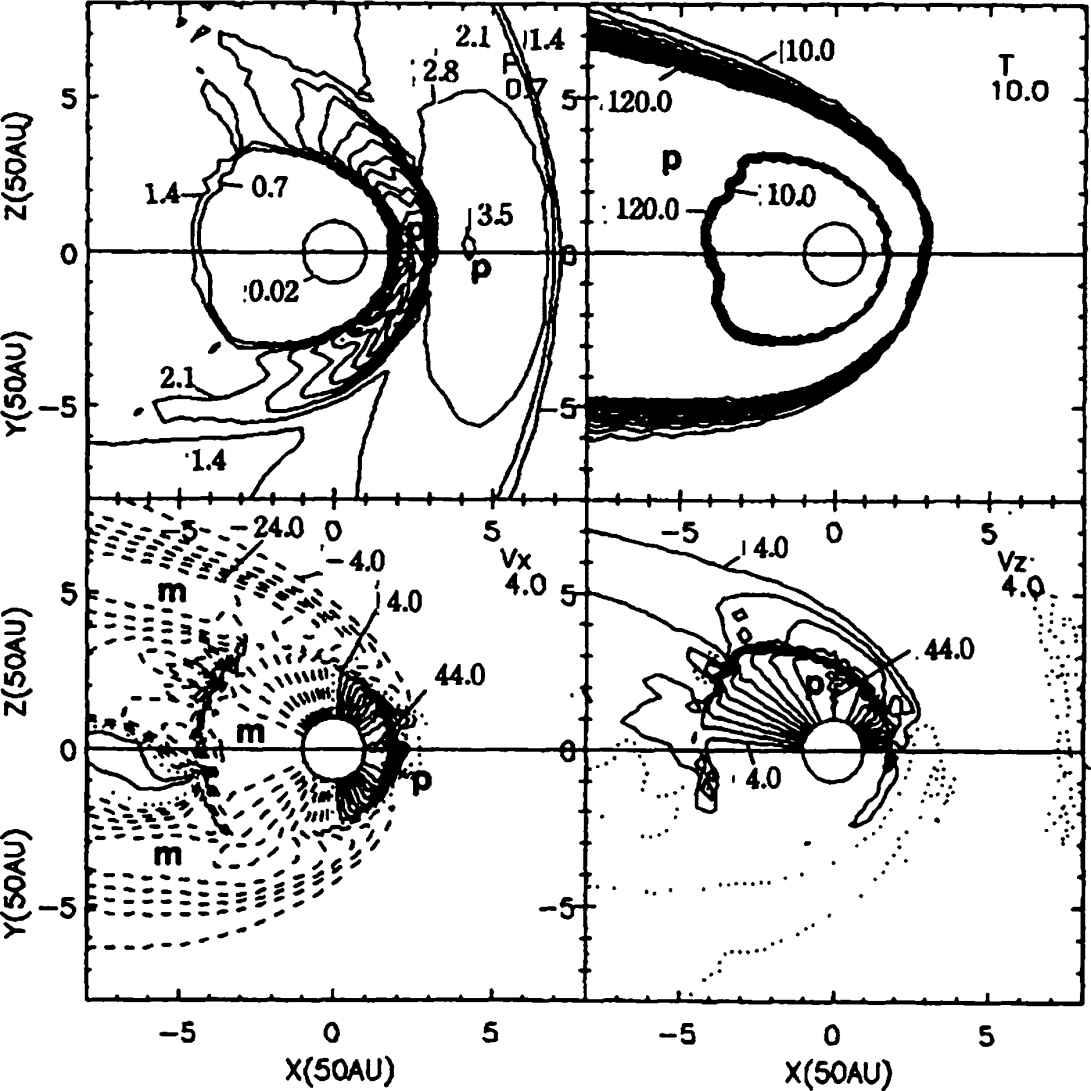
Global structure of the 3D MHD solar wind-LISM interaction. The upper and lower halves of each panel correspond to the meridional $(x,z)$ and equatorial $(x,y)$ planes respectively. From the upper left in clockwise order, the panels show pressure, temperature, $u_{z}$ and $u_{x}$ velocity contours respectively. Solid and dashed lines correspond to positive and negative values. The normalized values of pressure, temperature, and velocity are $1.44 \times 10^{-14}~\text{N}\,\text{m}^{-2}$, $10^{4}~\text{K}$, and $9.1 \times 10^{3}~\text{m}/\text{s}$. Regions of positive and negative maximum/minimum values are indicated by a $p$ or $m$ (Washimi and Tanaka 1996)

**Fig. 13:**
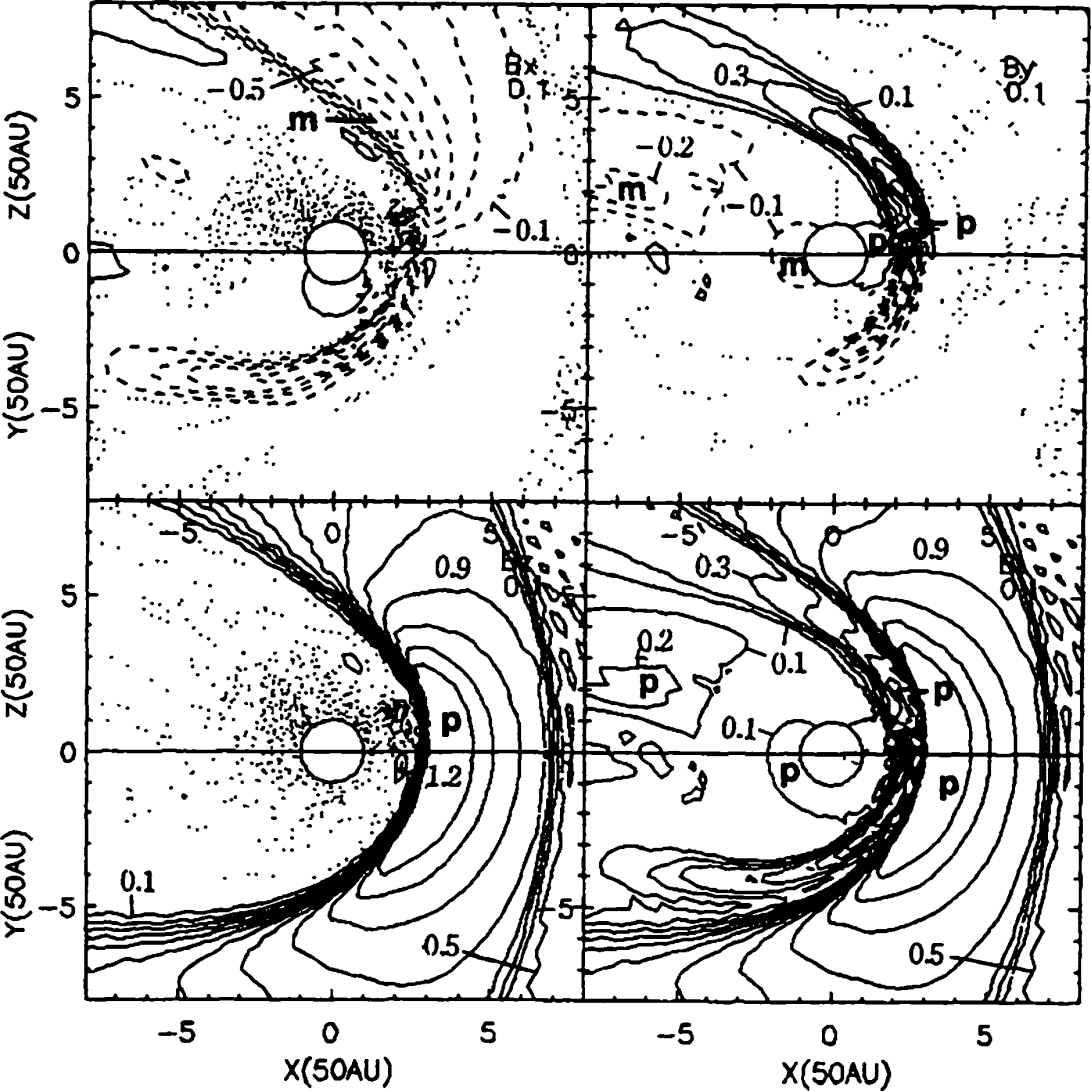
The global structure of the magnetic field corresponding to Fig. 12. The upper and lower halves of each panel correspond to the meridional $(x,z)$ and equatorial $(x,y)$ planes respectively. In clockwise order, the panels show $B_{x}$, $B_{y}$, $|{\mathbf{B}}|$, and $B_{z}$ contours respectively. The normalized value of the magnetic intensity in these panels is 0.3 nT. Solid and dashed lines correspond to positive and negative values. Dotted lines depict zero values of a variable. Regions of positive and negative maximum/minimum values are indicated by a $p$ or $m$ (Washimi and Tanaka 1996)

The LISM magnetic field in the Washimi and Tanaka (1996) simulations is perpendicular to the LISM flow and parallel to the solar rotation axis. The global structure is illustrated in Fig. 12, and the bow shock, heliopause and termination shock are evident. The characteristic bullet-shape of the HTS seen in the gas dynamic simulations is recovered. In Fig. 13, the magnetic field components and magnitude are plotted. The interplanetary toroidal component $B_{y}$ is enhanced in the heliosheath. An interesting result is that the neutral sheet in the heliosheath is bent upwards, so that the amplitude of $B_{y}$ is greater on the equatorial than on the meridional plane. The equipressure contours are a minimum in the middle region of the upstream heliosheath and the pressure depression is more evident on the equatorial than on the meridional plane. The depressions are due to a magnetic pressure effect and the pressure depletion in Fig. 12 coincides exactly with the $B_{y}$ enhancements of Fig. 13. This corresponds to the formation of a 3D magnetic shell or wall in the outer regions of the upstream heliosheath, a structure first identified by Washimi and Tanaka (1996). The outward flow of the shocked solar wind (the $u_{x}$ component) is rapidly curtailed, indicating that the magnetic wall in the middle and high latitudes diverts the flow tailward. Only on the upwardly distorted heliospheric current sheet is $u_{x}$ positive almost all the way to the heliopause. Thus, Washimi and Tanaka (1996) find that the region between the HTS and the magnetic wall is occupied by solar wind originating from the middle and high latitudes whereas the region between the magnetic wall is composed of solar wind originating from the equatorial neutral sheet region.

### The Inclusion of Neutral H in Global Models

The weak coupling of neutral gas and plasma (which provides an effective volumetric force) affects both distributions in important ways, and this coupling is crucial in modeling the interaction of the solar wind with the LISM. Baranov and Malama (1993, 1995), developed such a coupled model using a Monte-Carlo algorithm to evaluate the neutral H distribution and a 2D steady-state fluid description of the plasma. An alternative series of models, based on a multi-fluid description of neutral H was developed initially by Zank and his collaborators (Pauls et al. 1995; Zank et al. 1996d,c; Williams et al. 1997; Pauls and Zank 1997; Liewer et al. 1996). The need for modeling the neutrals as a multi-fluid stems from the variation in the charge exchange mean-free-path for H in different regions of the heliosphere and LISM. Large anisotropies are introduced in the neutral gas distribution by charge exchange with the solar wind plasma (both sub- and supersonic) and the multi-fluid approach represents an attempt to capture this characteristic in a tractable and computationally efficient manner.

The heliosphere-LISM environment can be described as three thermodynamically distinct regions; the supersonic solar wind (region 3), the very hot subsonic solar wind (region 2), and the LISM itself (region 1). Each region is a source of neutral H atoms whose distribution reflects that of the plasma distribution in the region. Accordingly, Zank et al. (1996d) identified neutral components 1, 2, and 3 with neutral atoms originating from regions 1, 2, and 3. Each of these three neutral components is represented by a distinct Maxwellian distribution function appropriate to the characteristics of the source distribution in the multi-fluid models. This observation allows the use of the simpler production and loss terms for each neutral component (Pauls et al. 1995; Zank et al. 1996d). The complete highly non-Maxwellian H distribution function is then the sum over the three components. Under the assumption that each of the neutral component distributions is approximated adequately by a Maxwellian, one obtains an isotropic hydrodynamic description for each neutral component,

55 $$\begin{aligned} \frac{\partial \rho _{i}}{\partial t} + \nabla \cdot \left ( \rho _{i} {\mathbf{u}}_{i} \right ) &= Q_{\rho i} ; \end{aligned}$$

56 $$\begin{aligned} \frac{\partial}{\partial t} \left ( \rho _{i} {\mathbf{u}}_{i} \right ) + \nabla \cdot \left [ \rho _{i} {\mathbf{u}}_{i}{\mathbf{u}}_{i} + p_{i} {\mathbf{I}} \right ] &= {\mathbf{Q}}_{mi} ; \end{aligned}$$

57 $$\begin{aligned} \frac{\partial}{\partial t} \left ( \frac{1}{2} \rho _{i} u_{i}^{2} + \frac{p_{i}}{\gamma - 1}\right ) + \nabla \cdot \left [ \frac{1}{2} \rho _{i} u_{i}^{2} {\mathbf{u}}_{i} +\frac{\gamma}{\gamma - 1} {\mathbf{u}}_{i} p_{i} \right ] &= Q_{ei} . \end{aligned}$$

The source terms $Q$ are appropriate moments of the source terms and are listed in Pauls et al. (1995), Zank et al. (1996d). The subscript $i$ above refers to the neutral component of interest ($i =1,2,3$), $\rho _{i}$, ${\mathbf{u}}_{i}$, and $p_{i}$ denote the neutral component $i$ density, velocity, and isotropic pressure respectively, ${\mathbf{I}}$ the unit tensor and $\gamma $ ($= 5/3$) the adiabatic index.

The plasma is described similarly by the 2D hydrodynamic equations

58 $$\begin{aligned} \frac{\partial \rho}{\partial t} + \nabla \cdot \left ( \rho {\mathbf{u}} \right ) &= Q_{\rho p} ; \end{aligned}$$

59 $$\begin{aligned} \frac{\partial}{\partial t} \left ( \rho {\mathbf{u}} \right ) + \nabla \cdot \left [\rho {\mathbf{u}}{\mathbf{u}} + p {\mathbf{I}} \right ] &= {\mathbf{Q}}_{m p} ; \end{aligned}$$

60 $$\begin{aligned} \frac{\partial}{\partial t} \left ( \frac{1}{2} \rho u^{2} + \frac{p}{\gamma - 1}\right ) + \nabla \cdot \left [ \frac{1}{2} \rho u^{2} {\mathbf{u}} +\frac{\gamma}{\gamma - 1} {\mathbf{u}} p \right ] &= Q_{e p} , \end{aligned}$$

where $Q_{(\rho ,m,e),p}$ denote the source terms for plasma density, momentum, and energy. They too are listed in Pauls et al. (1995), Zank et al. (1996d). The remaining symbols enjoy their usual meanings. The proton and electron temperatures are assumed equal in the multi-fluid models. It is important to remind the reader that the PUI component created by the charge-exchange process is included in the pressure term $p$ in the plasma equations (58)–(60).

The coupled multi-fluid system of Eqs. (55)–(60) are solved numerically. Pauls et al. (1995) use a two-fluid reduction of the Eqs. (55)–(60) in that components 2 and 3 are neglected entirely. This simplified approach has the virtue of computational efficiency while still retaining the basic features of the full model. The complication in the multi-fluid models lies in the correct identification of the boundaries (the HP and HTS) of the three (or four) thermodynamic regions (see Pauls et al. 1995; Zank et al. 1996d).

**Fig. 14:**
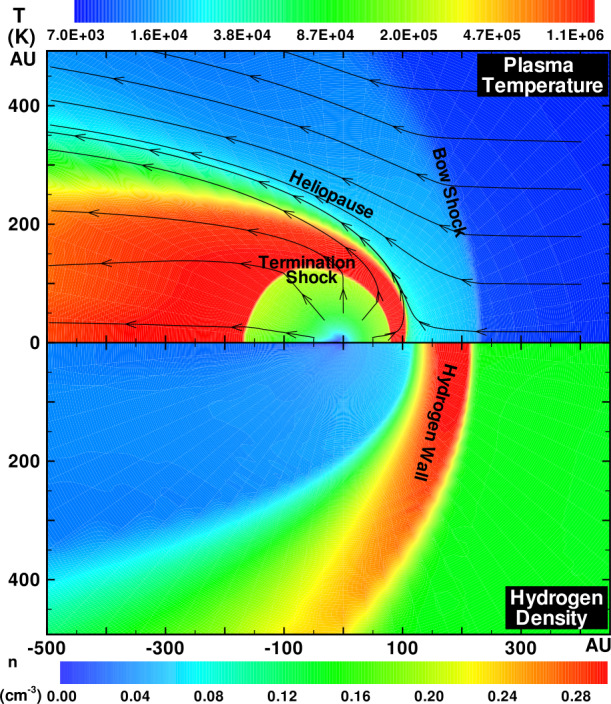
The 2D steady-state, 2-shock heliosphere showing, top plot, the temperature distribution in color of the solar wind and interstellar plasma and, bottom plot, the density distribution of neutral hydrogen. The plasma boundaries, termination shock, heliopause, and bow shock are labelled, and the wall of neutral hydrogen is also identified. The solid lines of the top plot show the streamlines of the plasma. The plasma temperature is plotted logarithmically and the neutral density linearly. The distances along the $x$ and $y$ axes are measured in astronomical units (AU) (Zank et al. 1996d; Zank and Müller 2003)

Consider the case of a supersonic interstellar wind incident on the heliosphere. Figure 14 (top) shows the normalized density, flow direction and temperature (color plot) for the $\text{H}^{+}$ fluid at steady-state, now including the effects of charge exchange that couples the neutral H gas and plasma. The positions of the three interfaces are indicated on the plot. A major effect of charge exchange on the heliospheric interfaces is to decrease the distances to the HTS, HP and BS. The reason for the decrease in distance results primarily from the decrease in the solar wind ram pressure, this due to the mediation of the supersonic solar wind by charge exchange.

Besides the distance to the various heliospheric boundaries, charge exchange effects the shape of the termination shock, making it more spherical than the purely gas dynamic model. This is due to charge exchange in the heliosheath, which decelerates the shocked solar wind plasma in this region. Thus, the heliosheath flow remains subsonic throughout the region and the need for a triple umbilic point disappears when the neutral hydrogen density is finite. Owing to the deposit of interstellar protons in the solar wind when charge exchange is included, the solar wind flow now departs slightly from simple spherical symmetry. The departure can scarcely be seen in Fig. 14 (top) but a comparison of Figs. 8 and 14 (top) shows clearly the contribution to the internal energy of the supersonic solar wind by pickup ions. The tail region is also cooler when the proton fluid and neutral fluid are coupled compared to the no-charge exchange model. The cooling is a consequence of the very hot heliotail being cooled by charge exchange with cooler component 1 neutrals.

**Fig. 15:**
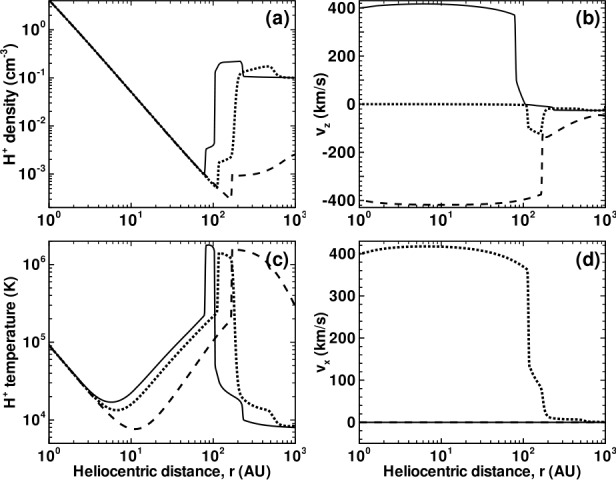
Lines-of-sight profiles for the two-shock model plasma in the upstream direction (solid line), side-stream direction (dotted line) and downstream direction (dashed line). Shown are (**a**) the density profiles; (**b**) the plasma velocity $v_{z}$; (**c**) the temperature, and (**d**) the velocity $v_{x}$ profiles (Zank et al. 1996d)

**Fig. 16:**
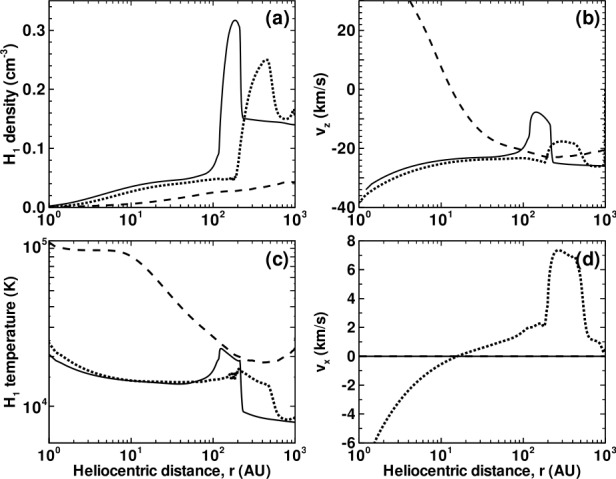
Lines-of-sight profiles for the two-shock model component 1 neutrals in the upstream direction (solid line), side-stream direction (dotted line) and downstream direction (dashed line). Shown are (**a**) the density profiles; (**b**) the plasma velocity $v_{z}$; (**c**) the temperature, and (**d**) the velocity $v_{x}$ profiles (Zank et al. 1996d)

Illustrated in Fig. 14 (bottom) is a 2D plot of the component 1 neutral distribution. Line-of-sight profiles for the plasma and component 1 neutrals are Shown in Figs. 15 and 16. The basic features can be summarized as follows. Inflowing component 1 neutrals are decelerated substantially and filtered by charge exchange with the interstellar plasma between the BS and HP in the upstream direction. This leads to the formation of a hydrogen wall with maximum densities $\sim 0.3~\text{cm}^{-3}$, column densities $\sim 10^{14}~\text{cm}^{-2}$ and temperatures ranging from 20,000 K to 30,000 K. The pile-up in the neutral gas results from the deceleration and deflection of the neutral flow by charge exchange with the interstellar plasma, which is itself decelerated and diverted due to the presence of the bow shock ahead of the heliosphere. Note that the charge exchange mean-free-path is typically less than the separation distance between the heliopause and bow shock and so a large part of the incident interstellar neutrals experience charge exchange. Component 2, produced via charge exchange between component 1 and hot shocked solar wind plasma between the HTS and HP, streams across the HP into the cooler shocked interstellar gas and heats the interstellar plasma through a secondary charge exchange (Zank et al. 1996d). This leads to an extended thermal foot abutting the outside edge of the HP. This heating of the plasma by component 2 serves to broaden the region between the BS and HP, as well as to (indirectly) further heat the component 1 interstellar neutrals after subsequent charge exchanges. Some minor heating of the unshocked LISM also occurs upstream of the BS, thereby marginally reducing the Mach number of the incident interstellar wind.

The temperature of component 1 neutrals once inside the heliosphere remains fairly constant in the upstream region, at $T \sim 20{,}000~\text{K}$, a substantial increase over the assumed LISM temperature of $\sim 7000~\text{K}$ assumed for Model 1. A further increase in the component 1 temperature occurs in the downstream region. The number density of component 1 crossing the HTS is $\sim 0.07~\text{cm}^{-3}$. This is approximately half the assumed incident LISM number density, an effect termed “filtration.” Between the HTS and 10 AU from the Sun in the upstream region, this density varies only weakly, following a rough power law ($\sim r^{0.25}$, with $r$ the heliospheric radius). In the downstream direction, component 1 densities are lower within the heliosphere and the gradient is somewhat more pronounced, with density increasing as $r^{0.35}$. The precise value of the component 1 density at the HTS can vary with parameters and an example is presented below. A further effect of filtration is to decelerate the upstream neutral gas from $-26~\text{km}/\text{s}$ in the LISM to $-19~\text{km}/\text{s}$ at the HTS in the region of the nose. Deflection of the flow also reduces the radial velocity component at angles away from the nose. Such a deceleration is in accord with Lyman-$\alpha $ backscatter observations (Lallement 1996; Quemerais et al. 1995).

Zank et al. (1996d) and Liewer et al. (1996) noticed from their simulations that the HP may be time dependent due to an inwardly directed ion-neutral drag term which provides an effective “gravitational” term for a stratified fluid (which then introduces the possibility of Rayleigh–Taylor-like instabilities). A time scale of $\sim 180$ years and an amplitude of $\sim 3~\text{AU}$ for the oscillations was found in these preliminary models. The theory was subsequently developed by Zank (1999b) and more detailed simulations subsequently confirmed the nature of the instability of the heliopause (Florinski et al. 2005; Borovikov et al. 2008).

The 3D gas dynamic model of Pauls and Zank (1996) was extended (Pauls and Zank 1997) to include interstellar neutral H in the multi-fluid framework. In modeling the neutral component, the simpler two-fluid approach of Pauls et al. (1995) was adopted, this to circumvent the computational demands imposed by the 3D simulation. Pauls and Zank (1997) use the same boundary conditions as used in their 3D gas dynamic study (Pauls and Zank 1996).

**Fig. 17:**
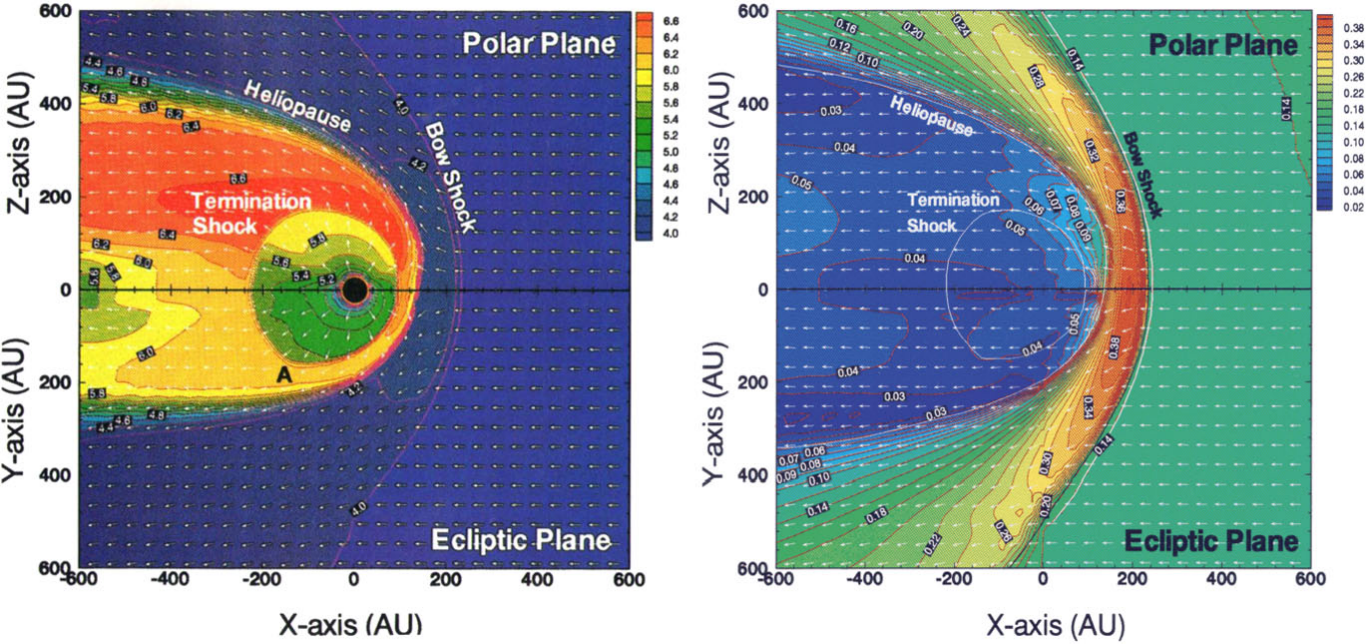
(Left) Log[Temp] (contour and color) and normalized flow vectors as a function of distance in the polar plane (top panel) and in the ecliptic plane (bottom panel). A denotes the position of a triple point. (Right) Interstellar neutral hydrogen density ($\text{cm}^{-3}$) (contour and color) and normalized flow vectors as a function of distance in the polar plane (top panel) and in the ecliptic plane (bottom panel). The positions of the plasma boundaries are indicated (Pauls and Zank 1997)

Figure 17 shows the normalized flow vectors and the $\log(\text{H}^{+}\text{ temperature})$ contours of the steady state solution as a function of distance. The top panel shows a cut through the three-dimensional data set in the polar plane, while the bottom panel is a cut in the ecliptic plane. The distances to the boundaries (HTS, HP, and BS) are again greatly reduced by charge exchange. The decrease in distance to the HTS is due mainly to the reduction in solar wind ram pressure associated with the deceleration of the supersonic wind by charge exchange.

An interesting result, illustrated in Fig. 17, is the presence of a triple point (letter A on the figure) attached to the termination shock in the ecliptic plane. Only for a 2D gas dynamic simulation of a supersonic LISM interacting with the solar wind does a triple point occur. However, the same 2D simulation with the self-consistent inclusion of interstellar neutrals does not have a triple point since the heliosheath flow never attains supersonic speeds. However, the elongation of the heliosphere over the poles of the sun causes the flow to be focussed in the ecliptic plane, both with and without the inclusion of interstellar neutrals. Thus, the ecliptic plane heliosheath flow speed is higher (high enough for the flow to become supersonic, so necessitating a triple point) than the flow speed over the poles. Figure 17 also shows cooling of the heliotail region. The elongation of the heliosphere along the solar poles leads to a larger density shocked LISM flow in the ecliptic plane compared to that over the poles. This 10% increase in plasma density is evident between the HP and BS when the two side-stream flows are compared (Pauls and Zank 1997). Since the charge exchange source terms are proportional to the plasma density, charge exchange is enhanced between the HP and BS in the ecliptic plane compared to the polar plane. This, in turn, causes a greater deceleration of the neutral H fluid in the ecliptic plane compared to the polar plane, and leads to a denser H pileup in the ecliptic plane compared to the solar poles. Since the density of the hydrogen wall is a maximum in the ecliptic plane, one has more effective neutral H filtration here. Thus, less interstellar neutral H can be expected to flow into the heliosheath in the ecliptic plane than at high solar latitudes. Besides the enhancement of the LISM flow in the ecliptic region, the shocked solar wind too is diverted into the ecliptic plane. This, together with the assumed decrease in supersonic solar wind density with increasing heliolatitude, leads to increased charge exchange in the ecliptic plane in the heliosheath (not enough, however, to prevent the ecliptic flow from becoming supersonic). Thus, less interstellar H flows into the supersonic solar wind in the ecliptic regions compared to the polar regions. The higher H density over the poles of the sun reduces the elongation of the HTS in the charge exchange case compared to the no-charge-exchange case (a 33% decrease in distance to the HTS over the poles, compared to a 29% decrease in the ecliptic plane when charge exchange is included in the 3D model). Thus, in summary, the elongation of the heliosphere over the solar poles, caused by a ram pressure increase in this region, results in an increased flux of interstellar H flowing into the heliosphere in the high heliolatitude regions compared to the ecliptic plane. This decreases, in turn, the elongation of the heliosphere over the poles. If, in fact, the neutral interstellar hydrogen density were much higher, the heliosphere might in fact revert to a more spherical structure.

One should ideally calculate the interstellar neutral distribution at a kinetic level since the Knudsen number $Kn \simeq 1$ for neutral H. Neutral hydrogen gas is coupled to the plasma through appropriate source terms. When a fluid description for the neutrals is assumed, source terms are calculated using approximations to integrals over the distribution functions convolved with the charge-exchange cross-section. Coupling a Monte-Carlo code to a plasma code is more difficult than the corresponding coupling to a multi-fluid model. To avoid making assumptions on the nature of the distribution function, source terms for the plasma must be gathered in terms of individual charge-exchange events. Each event contributes to the plasma source at one location. It therefore requires a large number of charge-exchange events within each grid-cell in order to generate smooth and accurate sources for the plasma. When solving a steady-state problem, we may simply compute as many particle trajectories through the domain as is necessary for smooth sources. Time-dependent problems may be solved by collecting sources over a time interval which is shorter than the shortest time-scales we are trying to resolve. Under this constraint, a large number of particles are needed typically to retain accuracy if the timescales present in the solution are small.

Extending the original hydrodynamic-like model of Baranov et al. (1981), Baranov and Malama (1993, 1995) used a Monte-Carlo approach to solve the neutral H Boltzmann equation and coupled this to a steady-state 2D hydrodynamic model of the solar wind and LISM plasma. The method of coupling is that of “global iterations.” Here the plasma code is run to a steady-state iteratively, using the source term generated by running the neutrals on the preceding plasma state until successive states converge.

Baranov and Malama (1993) solve the following steady-state gas dynamic equations, using a shock fitting method,

61 $$\begin{aligned} \nabla \cdot \rho {\mathbf{u}} =& 0 ; \\ \rho {\mathbf{u}} \cdot \nabla {\mathbf{u}} + \nabla p =& \rho {\mathbf{F}}_{1} \left ( f_{H}({\mathbf{x}}, {\mathbf{v}}), \rho , {\mathbf{u}},p \right ) ; \\ \nabla \cdot \left [ \rho {\mathbf{u}} \left ( \frac{u^{2}}{2} + \frac{\gamma}{\gamma -1} \frac{p}{\rho} \right ) \right ] =& F_{2} \left ( f_{H}({\mathbf{x}}, {\mathbf{v}}), \rho , {\mathbf{u}},p \right ) , \end{aligned}$$

where $f_{H}({\mathbf{x}}, {\mathbf{v}})$ is the neutral H distribution function. Baranov and Malama (1993, 1995) compute neutral H trajectories in the solar wind and LISM plasma using a trajectory splitting Monte-Carlo method (Malama 1991), from which the source terms ${\mathbf{F}}_{1}$ and $F_{2}$ can be evaluated. The trajectory splitting Monte-Carlo approach of Malama (1991) is equivalent to solving the Boltzmann equation (1) with the integral production and loss terms (2), (5) and (7). It is assumed that the plasma can be described as a Maxwellian distribution $f_{p} ({\mathbf{x}}, {\mathbf{v}})$ with respect to the gas dynamic values determined by (61). The source terms in (61) are then computed from equations (2), (5), and (7) after integrating over the neutral H velocity space. The gas dynamic equations (61) and the Boltzmann equation (1) are then solved iteratively until the solution converges. The distant LISM neutral H distribution is assumed to be Maxwellian. Besides charge exchange and photoionization, Baranov and Malama (1996) include electron impact ionization in the region between the termination shock and heliopause.

**Fig. 18:**
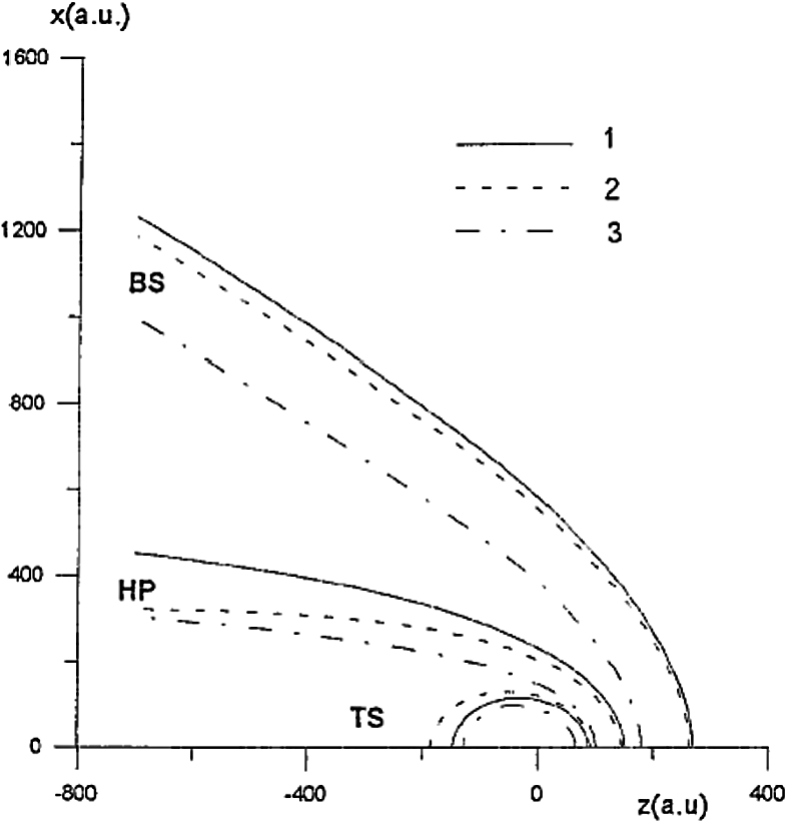
The boundaries of the termination shock, heliopause, and bow shock for undisturbed LISM plasma and neutral H densities (1) $n_{p} = 0.1~\text{cm}^{-3}$, $n_{H} = 0.2~\text{cm}^{-3}$, (2) $n_{p} = 0.1~\text{cm}^{-3}$, $n_{H} = 0.2~\text{cm}^{-3}$, and (3) $n_{p} = 0.3~\text{cm}^{-3}$, $n_{H} = 0.3~\text{cm}^{-3}$. Curves (1) and (2) differ in that (1) includes the effects of electron impact ionization (Baranov and Malama 1996)

Illustrated in Fig. 18 are three possible solutions for the global heliospheric structure obtained by Baranov and Malama (1993, 1996). Examples 1 and 3 correspond to models that incorporate the effects of resonant charge exchange, photoionization, gravity, and electron-impact ionization. Model 2 excludes electron-impact ionization and photoionization but is otherwise identical to model 1. The ratio of solar radiation pressure to gravitational force is $\mu = F_{r}/F_{g} = 0.8$.

**Fig. 19:**
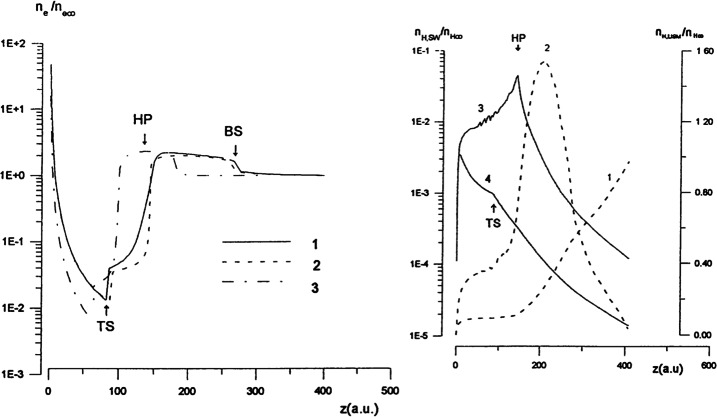
(Left) Plasma number density (normalized to that of the undisturbed LISM) as a function of heliocentric radius for the three different models of Fig. 18. (Right) Neutral number density as a function of heliocentric distance for $n_{p} = 0.1~\text{cm}^{-3}$, $n_{H} = 0.2~\text{cm}^{-3}$ in the upwind direction. The dashed lines are for neutral H whose source is the LISM (curve 1 is for primary LISM H and curve 2 for secondary LISM H i.e.,component 1) and the solid lines are for neutral H whose source is the solar wind (curve 3 is for component 2 H and curve 4 for component 3 H) (Baranov and Malama 1996)

The solutions illustrated in Fig. 18 show that the HTS in the upstream direction lie in the range 65–85 AU and some differences exist between the models that include or exclude electron-impact ionization. These pertain primarily to the location of the termination shock, indicating that in the heliosheath, electron-impact ionization may play an important role. Of course, given the nature of the shock heating of electrons and protons, it is unclear whether a Maxwellian description of the shocked solar wind plasma is entirely appropriate and this may well alter the quantitative conclusions that one draws from Fig. 18. One-dimensional profiles of the number density in the upstream direction for each of models 1, 2, and 3 are presented in Fig. 19 (left) and the basic characteristics have been discussed at length already.

The neutral H number density in the upstream direction is illustrated in Fig. 19 (right) for model 1. Four sets of curves are plotted, the dashed curves corresponding to interstellar neutral H and the solid curves to H born in the solar wind. Curve 1 denotes primary LISM H atoms and curve 2 is for the secondary atoms. Curve 1 illustrates clearly how the incident interstellar H atoms are depleted strongly by charge exchange in the region between the bow shock and heliopause. These H atoms are replaced by secondary atoms, shown by curve 2 in Fig. 19 (right), whose characteristics are determined by the shocked interstellar plasma. This leads, as discussed above, to the formation of the hydrogen wall, which consists of those neutral H atoms which experience charge exchange upstream of the HP. Curve 3 shows the number density of component 2 neutrals and curve 4 of component 3 neutrals. The energetically important component 2 neutrals peak in the vicinity of the HP and experience secondary charge exchange as they stream into the LISM, significantly heating the LISM plasma in the immediate vicinity of the HP.

Comparisons of the multi-fluid and kinetic neutral H approaches have since been presented (Alexashov and Izmodenov 2005; Heerikhuisen et al. 2006; Müller et al. 2008).

Linde et al. (1998) included interstellar neutral hydrogen in their 3D MHD simulations and investigated three possible LISM magnetic field orientations. The neutral H model is very simple in that it is assumed that the neutral H velocity and temperature remain constant and that interstellar H is lost only inside the HP. This then allows the solar wind to be mediated by the interstellar medium, so leading to a reduction in solar wind ram pressure. Such a neutral model clearly cannot reproduce the complicated neutral physics described by the gas dynamic models above, but it has the virtue of being relatively inexpensive computationally. The three LISM magnetic configurations considered by Linde et al. (1998) are ${\mathbf{B}}_{\mathrm{LISM}} \parallel {\mathbf{u}}_{\mathrm{LISM}}$ and ${\mathbf{B}}_{\mathrm{LISM}} \perp {\mathbf{u}}_{\mathrm{LISM}}$, the latter case with ${\mathbf{B}}_{\mathrm{LISM}} $ either in the equatorial plane or anti-parallel to the solar rotation axis. The last case corresponds to the model of Washimi and Tanaka (1996).

**Fig. 20:**
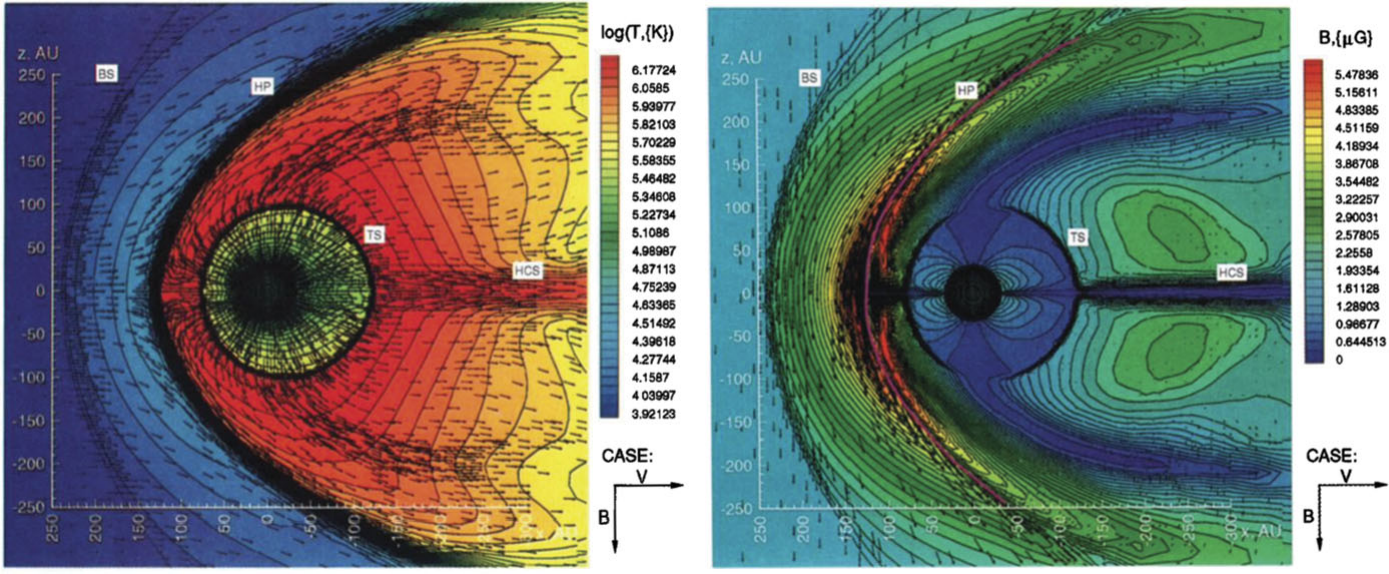
Model ${\mathbf{B}}_{\mathrm{LISM}} \perp {\mathbf{u}}_{\mathrm{LISM}}$. (Left) Contours of the Log[plasma temperature (K)] along the meridional plane. Small arrows indicate the plasma velocity field. (Right) Contours of the total magnetic field intensity along the meridional plane. Small arrows indicate the magnetic field direction (Linde et al. 1998)

To evaluate the role of the LISM mediated solar wind more clearly, we compare the results from the final case considered by Linde et al. (1998), i.e., ${\mathbf{B}}_{\mathrm{LISM}} \perp {\mathbf{u}}_{\mathrm{LISM}}$ and anti-parallel to the solar rotation axis, to those of Washimi and Tanaka (1996). Meridional cuts of the plasma temperature and magnetic field intensity are shown (Figs. 20 top and bottom respectively). Comparison of Figs. 12 and 20 (top) shows that the heliosheath flow remains subsonic when neutrals are included, and the bullet-shaped HTS becomes more spherical, consistent with the gas dynamic simulations. The magnetic wall is clearly present in Fig. 20 (bottom) (in fact, two walls). Magnetic reconnection appears to occur where the magnetic field has to re-orient across the HP. Interestingly, the tilt of the neutral sheet appears to be less pronounced in the simulations of Linde et al. (1998) than in those of Washimi and Tanaka (1996). Finally, the minimum distance to the HP occurs when the LISM magnetic field is perpendicular to the flow direction.

### Inferred Structure of the Heliosphere Circa 1996–1997

At a 1996 COSPAR meeting held in Boulder, CO, USA, J. Linsky heard a presentation given by G.P. Zank describing the structure of the heliosphere and the properties of the predicted hydrogen wall, borrowed his viewgraphs, and urged Zank to be in the audience the following day. The following morning, Linsky showed Lyman-$\alpha $ observations in the direction of the binary star system $\alpha $-Cen obtained by Hubble Space Telescope (HST) Goddard High Resolution Spectrograph (GHRS), with the interpretation that the red-shifted excess absorption was due to the presence of the H-wall. This was the discovery of the first of the boundary regions between the heliosphere and the LISM.

**Fig. 21:**
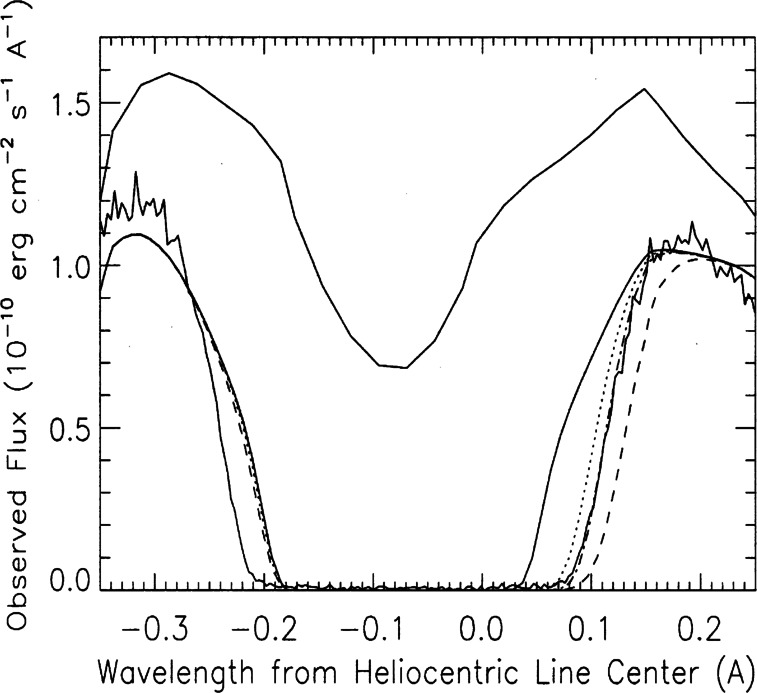
The solid curve is the GHRS Lyman-$\alpha $ profile towards $\alpha $ Cen A. The upper dashed curve is the assumed intrinsic stellar Lyman-$\alpha $ emission profile. The dotted curve shows the intrinsic stellar emission line after absorption by a purely LISM cloud with $N_{H} = 4.5 \times 10^{17}~\text{cm}^{-2}$, $b = 9.3~\text{km}/\text{s}$, and $v = -18.2~\text{km}/\text{s}$, with $\text{D/H} = 1.6\times 10^{-5}$ (Linsky and Wood 1996)

Linsky and Wood (1996) and Frisch et al. (1996) attributed the red-shifted excess absorption in GHRS Lyman-$\alpha $ observations towards $\alpha $ Cen (seen previously by Copernicus and IUE, Landsman et al. (1984)) to the solar hydrogen wall. Shown in Fig. 21 are the Linsky and Wood (1996) observations. The solid curves are the observed profiles for $\alpha $ Cen A and B, and the wavelength scale is relative to the Lyman-$\alpha $ line center in the heliocentric rest frame. The dashed curve is the assumed intrinsic stellar Lyman-$\alpha $ emission profile. In both the analysis of Linsky and Wood (1996) and Gayley et al. (1997) below, the accurate representation of the intrinsic stellar profile is unimportant since the absorption features of interest vary sharply. The dotted curves in Fig. 21 give the attenuation of the stellar Lyman-$\alpha $ emission by interstellar H with column depth $N =4.5 \times 10^{17}~\text{cm}^{-2}$, velocity $V = -18~\text{km}/\text{s}$ and a Doppler broadening $b = 9.3~\text{km}/\text{s}$. The value for $N$ was fixed by scaling to the Deuterium column density, assuming the commonly accepted value of $\text{D/H} = 1.6\times 10^{-5}$ (Linsky et al. 1995). Figure 21 shows very clearly that additional absorption is required both redward and blueward of the interstellar feature if the fit is to be completed. Furthermore, the fit must be applied preferentially to the redward side, so arbitrarily changing the D/H ratio is unacceptable. Linsky and Wood (1996) and Frisch et al. (1996) interpreted the additional redward absorption as evidence for the detection of the hydrogen wall associated with our heliosphere. The blueward absorption suggests the possibility of a hydrogen wall about $\alpha $ Cen A and B.

**Fig. 22:**
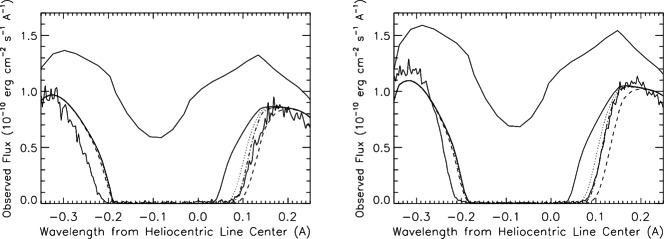
Similar to Fig. 21, except that absorption from three heliospheric models is included. All curves from Fig. 21 are reproduced as solid lines, while the dashed curve is for model 1 ($M=1.5$), dotted for model 2 ($M=0.7$), and dot-dashed for model 3 ($M=0.9$). Here, the left and right panels refer to $\alpha $ Cen A and B respectively. The red edge of the LISM absorption feature is fitted best by model 3, and observe that none of the models can fit the blue edge. This raises the possibility that $\alpha $ Cen A and B also possess a hydrogen wall (Gayley et al. 1997)

To see why the H wall should produce an observable signature in the Lyman-$\alpha $ absorption data, consider the optical depth of the H wall,

62 $$ \tau _{hw} (\lambda ) = {\frac{7.5 \times 10^{-13} }{b_{hw}}} N_{hw}e^{-(247 \lambda - v_{hw})^{2}/ b_{hw}^{2}} , $$

where $v_{hw}$ and $b_{hw}$ are in km/s (and negative $v_{hw}$ corresponds to motion toward the Sun), and $\lambda $ is in Å from line center in the heliocentric frame. The narrow absorption domain of interest in Fig. 22 appears in the vicinity of $+0.1$ Å, corresponding to a Maxwellian sub-population moving at $+25~\text{km/s}$ away from the Sun. Linsky and Wood (1996) achieved a reasonable fit to the profile in this domain using $N_{hw} = 3 \times 10^{14}~\text{cm}^{-2}$, $b_{hw} = 22~\text{km}/\text{s}$, and $v_{hw} = -8~\text{km}/\text{s}$. Using these parameters, equation (62) yields $\tau _{hw} = 1.12$ at $\lambda = +0.1$. Thus, a simple constraint is that any heliospheric model invoked to explain this absorption feature must yield an optical depth of roughly unity at $\lambda = +0.1$ in the heliocentric frame.

Since the column depth of the hydrogen wall is three orders of magnitude smaller than the column depth in the LISM toward $\alpha $ Cen, it may be surprising at first glance that the heliospheric optical depth at $+0.1$ Å is of the same order as the LISM optical depth at that wavelength. The key difference is that the hydrogen wall is heated and decelerated, which both broadens and redshifts the heliospheric component away from the −0.07 Å centroid of the LISM absorption and toward the $+0.1$ Å wavelength of interest. To compare the relative importance of the temperature increase and the velocity shift in allowing the hydrogen wall to be visible, one finds from equation (62) that decelerating the projected velocity of the hydrogen wall along the $\alpha $ Cen sightline by an additional 1 km/s (from $-8~\text{km}/\text{s}$ to $-7~\text{km}/\text{s}$) has the same effect as increasing the temperature by 2300 K (if $b_{hw}$ is purely thermal, so that $T_{hw} = 61 b_{hw}^{2}$). Each would increase the optical depth by 15%. Since the Linsky & Wood $v_{hw}$ is redshifted by 10 km/s relative to the LISM, and heated by about 24,000 K, extrapolating the above analysis suggests that each of these effects contributes about equally in making the hydrogen wall visible. However, the nonlinear response to temperature rapidly becomes important as the temperature falls, and equation (62) indicates that $\tau _{hw} (0.1)$ falls by a factor of 5 if $b_{hw}$ is reduced to 16 km/s, corresponding to $T_{hw} \simeq 16{,}000~\text{K}$. For this reason, the temperature is the parameter that shows the most significant variation, and is therefore the most critical discriminant.

Gayley et al. (1997) used equation (62) locally in conjunction with the model heliospheres of Zank et al. (1996d) and the Linsky and Wood (1996) assumptions about the interstellar H parameters to obtain synthetic Lyman-$\alpha $ absorption spectra. Figure 22 shows the Lyman-$\alpha $ absorption at the red edge of the LISM feature for three heliospheric models – a supersonic model with a sonic Mach number $M=1.5$, a barely subsonic model $M=0.9$, and a fully subsonic model $M=0.7$. The modeled results were then compared to the GHRS data of Linsky & Wood (the solid curve). The primary and very important conclusion to emerge from the Gayley et al. study is that the synthetic Lyman-$\alpha $ profiles supported the detection of the hydrogen wall by Linsky & Wood. Thus, it appears that the hydrogen wall was indeed observed! Comparing the results of models 1–3 with the observations demonstrates the following points, all of which are robustly insensitive to variations in the plausible intrinsic profile. (1) Heliospheric Lyman-$\alpha $ absorption in the highly supersonic model (model 1) is too strong due to the stronger deceleration and especially the increase in temperature of the interstellar neutrals in the hydrogen wall. (2) Heliospheric Lyman-$\alpha $ absorption in the subsonic model with low Mach number (model 2) is too weak, since the more gradually diverted interstellar plasma flow leads to less deceleration and less heating of the interstellar neutrals. (3) The model with a barely subsonic Mach number of 0.9 (model 3) and a larger plasma density does yield a more favorable fit, giving compression and charge exchange heating of the neutrals intermediate to the results of Models 1 and 2. The consistency with GHRS data given by the parameters of model 3 should not be expected to be unique, and other combinations may well suffice. Models describing the absorption of Lyman-$\alpha $ by the H-wall have become considerably more sophisticated with the development of ever more detailed heliospheric models (Zank et al. 2013; Wood et al. 2014). However, Gayley et al. (1997) suggest that the incident interstellar gas flow should be neither highly supersonic nor highly subsonic, since these possibilities lead rather inevitably to Lyman-$\alpha $ absorption that is either too strong or too weak respectively.

### Ulysses Observations of Galactic Cosmic Rays at the Heliospheric Poles

Galactic cosmic rays (GCRs), the bulk of which are likely produced by supernovae remnants, permeate the Galaxy and enter the heliosphere where they encounter the solar wind and its magnetic field. This topic is covered in the review by Rankin et al. (2022, this journal).

Fisk (1981) discussed the prediction for the flux of cosmic rays above the heliospheric poles in advance of the International Solar Polar Mission (ISPM). It was noted that the traditional argument, prior to the discovery of the importance of cosmic-ray drifts (Jokipii et al. 1977), was that the GCR flux at high heliographic latitudes would be considerably larger than near the heliographic equator, and possibly even resemble the pristine GCR flux in the local interstellar medium. The traditional argument was that because the magnetic field of the solar wind, the well-known Parker spiral field, is considerably weaker at high latitudes, galactic cosmic rays would have easier access to the heliosphere in these regions. In some sense, this acts like a “funnel”. Cosmic-ray drift motions, first emphasized by Jokipii et al. (1977), modify this simple picture, however. In fact, as noted in Fisk’s paper, if the ISPM mission explored these regions in 1988–1989, as initially intended, the GCR drift patterns at that time would significantly modulate the GCR fluxes above the poles. At this time, there remained some skepticism of the importance of drifts, and Fisk argued that it was vital to measure the fluxes of GCRs in the polar regions, providing a key scientific motivation for the ISPM mission.

As galactic cosmic rays enter the heliosphere, they are subject to a number of important transport effects which influences their global distribution. These include: advection with the solar wind, spatial diffusion, energy change, and drift motions. These are the four main effects that are included in the well-known Parker transport equation (Parker 1965). Spatial diffusion of GCRs arises from their interaction with magnetic-field turbulence (cf. Jokipii 1966, 1968; Hasselmann and Wibberenz 1970), while advection with the solar wind arises because the magnetic fields are assumed to be stationary in the solar wind frame of reference, and when an individual charged particle “scatters” in this turbulent field, its energy is conserved. Note that if the field is time dependent in the plasma frame, stochastic energy gains and losses may result, but these are generally ignored in the Parker transport equation. Energy change results from the GCR interaction with expansion and compression of the solar wind plasma. The most-important effect for GCR transport is that of adiabatic cooling in the expanding solar wind. For a review of this topic, see Jokipii (1971). Drifts are also known to be important, and are especially so in the context of this section, as we now discuss.

Jokipii and Thomas (1981) emphasized the importance of the heliospheric current sheet in the modulation of cosmic rays, and laid the foundation for our understanding of how the polarity of the solar magnetic field establishes the drift patterns of GCRs as they move throughout the heliosphere. During the phase of the solar cycle in which the magnetic field in the northern solar hemisphere points outward from the Sun, the GCR drift motions in either hemisphere are equator-ward. At the equator, the drift motions along the current sheet are radially outward. At the solar-wind termination shock, the drift motions are poleward, due to the gradient in the magnetic field at the shock, thus completing a “cycle” that resembles a GCR convection pattern. This phase of the solar cycle is known as “A positive”, or $\text{A}>0$. During this phase, cosmic rays generally enter the inner heliosphere from the poles of the heliosphere. It is during this phase that it was initially thought that an observer at high heliographic latitudes might observe a higher GCR flux compared to that at that the equator, as discussed above. In the opposite solar magnetic polarity, however, the drift motions are reversed, and GCRs reach the inner heliosphere primarily by drifting inwards along the heliospheric current sheet. Since the current sheet is “wavy”, like a ballerina skirt as it is sometimes called, the current sheet itself can cause a modulation of the GCRs. This was the phase of the solar cycle in which ISPM would have first sampled high heliographic latitudes, and drifts may have reduced the intensity of GCRs above the solar poles, as was mentioned in the Fisk (1981) paper on this topic.

ISPM was later renamed Ulysses, and was launched in 1990. An encounter with Jupiter was used to establish it into a polar orbit. It first went to a high southern latitude, and then, after a “fast latitude scan”, it passed into the northern solar hemisphere, making measurements at high northern latitudes. Simpson et al. (1995b,a) reported the first measurements of cosmic rays in this region finding that the latitudinal gradient of GCRs was much smaller than had been anticipated. The GCR fluxes at the poles of the heliosphere, where Ulysses measured them, were only somewhat larger than those at the equator. While this was noted as a major discovery at the time, it should be noted that Jokipii and Kota (1989) actually predicted this to be the case. This is because the interplanetary magnetic field contains large-scale transverse magnetic fields due to turbulence that effectively leads to the field being essentially normal to the radial direction far from the Sun. They noted that the transverse components of the magnetic field in a spherical geometry decay with distance as $1/r$, whereas the radial component decays as $1/r^{2}$. Large-scale turbulence, caused by the random plasma convective motions in the solar photosphere (e.g. Jokipii and Parker 1968), are one source of this turbulence. Jokipii and Kota (1989) used observed values of solar supergranulation to estimate the polar heliospheric magnetic field, and concluded that the nature of the field would be very close to normal to the radial direction far from the Sun. And, since the diffusion of cosmic rays across the magnetic field is considerably less efficient than that along the field (cf. Giacalone and Jokipii 1999, and references therein), GCR fluxes would be reduced considerably in the polar regions of the heliosphere, compared to that expected if the field were consistent with the simple Parker-spiral field representation, which was later confirmed by Simpson et al.’s observations.

### Interstellar Dust and Its Interaction with the Heliosphere

In 1976, Bertaux and Blamont (1976) compared observations to theoretical predictions that interstellar dust passing through the solar system should be focused “downstream” of the Sun with respect to the incident beam, as is the case of hydrogen and helium. However, they found that fewer particles were observed than predicted (i.e., a ‘line of focusing’ was not observed in the zodiacal light) and proposed several mechanisms for explaining this. Bertaux and Blamont (1976) concluded that interstellar dust was best measured by dust detectors in space. Soon thereafter, Levy and Jokipii (1976) offered an alternative explanation for the unobserved focusing line, namely that charged interstellar dust particles would be largely excluded from the solar system because of the Lorentz force exerted on the dust grains by the interplanetary magnetic and flow field. Three years later, Gustafson and Misconi (1979) and Morfill and Grün (1979) calculated the trajectories of charged interstellar dust particles, finding that dust grains would experience a ‘focusing phase’ and a ‘defocusing phase’ with solar cycle, depending on the polarity of the solar and thus the interplanetary magnetic field.

The first direct measurements of interstellar dust particles in the solar system were made by the Ulysses spacecraft, in 1993, using an impact ionization dust detector (Grun et al. 1993). Impact ionization detectors are based on the principle that dust particles vaporize and ionize (including material from the target itself) on impact with the detector target. The velocity with which the ions and electrons, separated by an electric field, reach the detectors provides an estimate of the impact velocity of the particles (albeit with a large uncertainty, which resulted in the development of later theoretical models to improve the determination of velocities). The individual mass of an impacting particle can estimated from the total measured charge after impact and the estimated impact velocity. The direction of the impact is constrained by the instrument field of view, which was $140^{\circ}$ for Ulysses ($180^{\circ}$ if wall impacts are also considered). Thereafter, Galileo, Helios and Cassini during its cruise phase, measured interstellar dust particles using an impact ionization dust detector (Baguhl et al. 1996; Altobelli et al. 2003, 2005, 2006). Dust particle properties, in particular solar radiation pressure efficiency, were constrained from the Ulysses observations combined with theoretical modeling of interstellar dust grain trajectories (Landgraf et al. 1999). Ulysses data were further analyzed and found to exhibit a solar-cycle dependence in the flux of particles, as predicted by the theoretical studies in the 1970s (Gruen et al. 1994; Baguhl et al. 1996; Landgraf et al. 2000; Krüger et al. 2007; Sterken et al. 2015). The 16 years worth of Ulysses interstellar dust (ISD) data provides the largest and most reliable dataset for studying the ISD-heliosphere interaction currently (Krüger et al. 2015; Strub et al. 2015; Sterken et al. 2015). Despite extensive analysis of Ulysses and the other ISD data and detailed simulations, Sterken et al. (2015) nonetheless found that only part of the Ulysses dataset could be explained using certain basic dust grain properties, and not the entire time-series. A possible explanation for this conundrum, they suggested, was to include the heliospheric interface (and the variation in dust grain charges) in the simulations. WIND data, measured by collisions of dust with the antennae, suggest a solar-cycle dependent variability in the flux of the dust grains (Malaspina and Wilson 2016). The comparison of infrared observations to modeled cometary, asteroidal and interstellar dust in Rowan-Robinson and May (2013) indicated that there is an isotropic foreground radiation due to interstellar dust and further ground-based kinematic studies concerning its variability with the solar cycle are needed.

Simulation of the dust-heliosphere interaction were performed from the 1990s, using Monte Carlo simulation codes (Landgraf 1998, 2000). These simulations were closely related to the datasets of the Ulysses and Galileo spacecraft in an effort to better understand the observed dust mass distributions, the possibility that interstellar dust is filtered as it enters the heliosphere, and how it responds to the solar cycle. Too few “small” particles (less than about 0.3 micrometer radius) were observed with respect to the inferred dust grain size distribution derived from astronomical observations (e.g. Mathis et al. 1977), possibly a consequence of dust grain filtering in the solar system and at the heliospheric boundary, and due to instrument sensitivity limitations. Dust flow and filtering characteristics were studied in detail at several locations in the solar system (Sterken et al. 2012b, 2013) and optimum phases were identified for future measurements, perhaps in 2030 (e.g. Sterken et al. 2012a). These models did not yet include the heliospheric boundary regions and assumed a constant particle surface potential, but they did include the time-variability of the interplanetary magnetic field during the dust particle travel time.

Kimura and Mann (1998) studied the charging mechanisms of interstellar dust, including in the heliosheath regions to find that the particle surface potential was nearly constant in the solar system, but much higher in the heliosheath and depends as well on particle composition (silicates have a higher surface potential than carbonaceous materials). Linde and Gombosi (2000) simulated dust trajectories at the heliospheric interface in a “defocusing” configuration of the magnetic fields, i.e. in the 1996 solar minimum. They conclude that particles are filtered by the interface, excluding primarily grains of 0.1 and 0.2 μm radius. Czechowski and Mann (2003b) explored how small dust particles (less than 0.2 μm) can enter the heliosphere, in particular during the solar minima that corresponds to the focusing phase when grains can penetrate deeper into the solar system via the wavy heliospheric current sheet. Subsequently, Czechowski and Mann (2003a) investigated very small (ca. 10 nm) dust particles outside the heliospheric (and astrospheric) boundaries. Landgraf (2006) considered dust distributions in and near the “paleoheliosphere” and concluded that a smaller heliosphere would have led to more dust in the interplanetary environment via secondaries from an increased “small interstellar dust” number flux. Using a full global heliospheric model, Slavin et al. (2012) used Monte Carlo simulations of grain trajectories in a heliosphere that included the heliospheric boundaries and variable dust particle potentials. However, the time-varying solar cycle change over the flight time of the dust particles in the Solar System was neglected. Typical dust grain velocities are ca. 26 km/s and distances to the heliopause vary depending on solar cycle phase. Dust particles entering the heliosphere can take as long as 20 years to reach the Sun and another 20 years or more to leave the heliosphere. In a (static) “focusing” configuration of the magnetic fields, particles of size greater than about 30 nm can enter the Solar System in these simulations, whereas in the “defocusing” configuration only particles larger than 0.3 μm can reach distances close to the solar system planets. Some particles (e.g. those of 0.2 μm) were diverted towards the northern and southern latitudes of the heliosphere in the Slavin et al. (2012) simulations. Similar features, although less far North and Southward from the Sun, were seen in the simulations in the solar system (Sterken et al. 2012b). Strub et al. (2019) improved the Landgraf (2000), Sterken et al. (2012b) models for faster computation times and hence, higher resolution, for use at 1 AU from the Sun. This model (ESA-IMEX-ISD) does not include either the possibility of a variable dust grain surface charge or the heliosphere interface, but it does include a time-dependent interplanetary magnetic field over time it takes for a dust grain to travel through the Solar System. Alexashov et al. (2016) modeled the interstellar dust distribution in a global heliosphere including the interface, using a 3D kinetic-MHD model of a similar kind as used by Slavin et al. (2012). Both simulations used a variable dust grain charge. A Lagrangian fluid approach to the problem was taken by Mishchenko et al. (2020), who described “peculiarities” in the dust flow, in particular identifying “caustics.” These simulations did not incorporate a temporal heliospheric magnetic field, and assumes a fixed dust grain potential of $+3~\text{V}$. Although some caustics were also present in some trajectory plots in Sterken et al. (2012b), they were less prominent because of the temporal interplanetary magnetic field during a grain’s travel time. Godenko and Izmodenov (2021) studied the effects of dust velocity dispersion on the distributions inside the heliosphere for this specific model. A recent review on interstellar dust in the solar system can be found in Sterken et al. (2019).

## The Spacecraft

Numerous spacecraft have provided the key observations that made the study of the interaction of the heliosphere with the LISM possible. Here, we describe a few of the primary missions, the critical instrumentation, the investigators, and in some cases, the particular observations.

**OGO-5**: The NASA OGO-5 spacecraft, the fifth of six Orbiting Geophysical Observatories, was launched in 1968 to conduct 25 geophysical experiments for a better understanding of the earth as a planet. Two of the OGO-5 were designed to measure Lyman-$\alpha $ emission from the geocorona. These were the European Geocoronal Lyman-Alpha Measurement instrument (PI: Jacques Blamont) and the US Ultraviolet Airglow instrument (PI: Charles Barth). To achieve a better understanding of the Lyman-$\alpha $ background, OGO-5 was placed in a special spinning mode on three occasions when well outside the geocorona. The measurement of the intensity of the 1216 Å Lyman-$\alpha $ emission from outside the geocorona resulted in the discovery of the interstellar wind by Bertaux and Blamont (1971) and Thomas and Krassa (1971).

**Pioneer 10 & 11**: Pioneer 10 and its twin probe, Pioneer 11, were the first spacecraft designed to explore the outer Solar System, including the interplanetary medium beyond the orbit of Mars. Pioneer 10 was launched on March 3, 1972 and was the first of five satellites (Pioneer 10 & 11, Voyager 1 & 2, and New Horizons) to achieve the escape velocity needed to leave the Solar System. The Pioneer 10 trajectory was towards the heliotail direction i.e., in the “downwind” direction. The Principal investigator on Pioneer 10 was Dr. James Van Allen. Communications with Pioneer 10 were lost on January 23, 2003 when at a distance of 80 AU. Because Pioneer 10 was flying toward the heliotail, Pioneer 10 was most likely not very close to the HTS. Pioneer 10 is the only spacecraft to give some insight into the distant downwind heliosphere but little related detailed modeling has been done using the Pioneer 10 observations (Nakanotani et al. 2020).

Pioneer 11 was launched on April 5, 1973, nearly a year later, with a trajectory toward the “nose” of the heliosphere – effectively in the opposite direction as Pioneer 10. By 1995, Pioneer 11 no longer had sufficient power for any of its instruments, with the last signal occurring on November 24, 1995. On January 30, 2019, Pioneer 11 was 100 AU from the Sun, suggesting that it had crossed the HTS at some point and was then in the inner heliosheath region.

The Pioneer 10 & 11 spacecraft carried instrumentation relevant to the outer heliosphere: a Helium Vector Magnetometer (HVM) (PI; E.J. Smith); a Quadrispherical Plasma Analyzer (PI: A. Barnes); the Charged Particle Instrument (CPI) (PI: J. Simpson); Cosmic Ray Telescope (CRT) (PI: F. McDonald); Geiger Tube Telescope (GTT) (PI: J. Van Allen); Ultraviolet Photometer (PI: D. Judge); and a Triaxial Fluxgate Magnetometer (PI: M. Acuna), that was carried only on Pioneer 11.

Pioneer 10 & 11 provided a trove of important energetic particle results, especially the discovery of the anomalous cosmic ray component, together with numerous important plasma, magnetic field, and Lyman-$\alpha $ measurements.

**SOHO**: Launched in December 1995, the joint NASA-ESA Solar & Heliospheric Observatory mission – SOHO – was meant to operate until 1998, but has had several mission extensions over the past two decades and multiple solar cycles. SOHO is equipped with 12 instruments of which the SWAN or Solar Wind ANisotropies instrument (PI: Formerly J-L. Bertaux and now E. Quemerais) has been critical to furthering our understanding of the interstellar wind as it drifts through the heliosphere. SWAN measures the Lyman-$\alpha $ light backscattered by neutral interstellar H and He drifting through the heliosphere – the Lyman-$\alpha $ resonance glow.

**The Voyager Interstellar Mission**: The twin NASA spacecraft Voyager 1 and 2, although launched on September 5 and August 20, 1977 respectively from Cape Canaveral, Florida aboard a Titan-Centaur rocket, only became the Voyager Interstellar Mission after the Voyager 2 flyby in 1989 of Neptune. At this time, Voyager 1 was $\sim 40~\text{AU}$ from the Sun and Voyager 2 $\sim 31~\text{AU}$. Voyager 1 is traveling northward at $35^{\circ}$ from the ecliptic plane, in the general direction of the Solar Apex (the direction of the Sun’s motion relative to nearby stars) at a speed of about 3.6 AU per year, whereas Voyager 2 is headed southward at $48^{\circ}$ from the ecliptic at a speed of about 3.3 AU per year.

The VIM PI is Edward C. Stone, and he is also the PI of the Cosmic Ray Sub-system (CRS), one of 5 identical instrument suites on both Voyager 1 & 2. The PI of the Low-Energy Charged Particles (LECP) suite is Stamatios (Tom) M. Krimigis, the Magnetometer (MAG) PI was Norman F. Ness and now Adam Szabo, the Plasma Science (PLS) was John Belcher and now John Richardson, and finally the PI of the Plasma Wave Subsystem (PWS) was Don Gurnett and now Bill Kurth. Unfortunately, the PLS instrument on Voyager 1 was damaged during the Jovian encounter. It is impossible to list the discoveries and achievements made by each of these Voyager instruments – all have redefined in one way or another our basic understanding and knowledge of the interaction of the heliosphere with the LISM.

For nearly 15 long years, the heliospheric community tried to read the “plasma tea leaves” in an effort to glean when a heliospheric termination shock crossing might occur (and to stave off congressional and other efforts to eliminate VIM funding!). The most popular estimate for the distance to the HTS that was polled during a series of University New Hampshire outer heliosphere meetings was 10 AU ahead of wherever Voyager 1 was! In some respects, this guess proved prescient since in the 4–5 years before Voyager 1 crossed the HTS at 94 AU in December 1994, Washimi et al. (2007) showed that the HTS did indeed move out with the increased ram pressure associated with the changing solar cycle, remaining roughly 5–10 AU ahead of Voyager 1. Voyager 2 crossed the HTS at 84 AU in August 2007 suggesting that the heliosphere was possibly quite asymmetric. Voyager 1 spent approximately 7 years exploring the inner heliosheath, a region that remains quite mysterious to this day (although see e.g., Decker et al. 2012), before reaching the HP at $\sim 122~\text{AU}$ and crossing into interstellar space on August 25, 2012. The entry of Voyager 1 into interstellar space was an historic occasion, the first human-made object to escape the confines of the heliosphere an astonishing 55 years after the space age began in 1957! Voyager 2 followed suit on November 5, 2018. Both the Voyager 1 & 2 crossings of the HP were associated with a virtual depletion of particles of solar origin at all levels, an abrupt increase of GCR, magnetic field and plasma density upwind at the HP, whereas the temperature was somewhat higher than expected. However, despite those similarities, some considerable differences were also identified (Krimigis et al. 2019) (see also Dialynas et al. 2022, this journal). Voyagers 1 & 2 are now exploring the very local interstellar medium *in situ*, continuing to make surprising, remarkable, and profound discoveries and measurements that will fuel our understanding of the interaction of the solar wind for the next decade despite having only enough electrical power and thruster fuel to keep its current suite of science instruments on until 2025 or a little later. The Voyagers manifest destiny is to wander the confines of our galaxy for all eternity. Voyager 1’s first encounter with another star will be in about 40,000 years when it will be within 1.6 light-years of AC+79 3888, a star in the constellation of Camelopardalis which is heading toward the constellation Ophiuchus. Also in about 40,000 years, Voyager 2 will pass within 1.7 light-years of the star Ross 248 and in about 296,000 years, it will pass within 4.3 light-years from Sirius, the brightest star in the sky.

**Ulysses**: The Ulysses mission resulted from a planned International Solar Polar Mission that would have included two spacecraft, one American and one European, flying over opposite solar poles to investigate the three dimensional Sun. Unfortunately, the NASA contribution was canceled and instead a single European Space Agency (ESA) spacecraft, Ulysses, was flown with a scientific payload shared by ESA and NASA. Ulysses flew a unique trajectory, using a gravity assist from Jupiter to take it below the ecliptic plane and past the solar South Pole and then above the ecliptic to fly over the North Pole.

Ulysses provided critical in situ observations of the 3D temporal solar wind structure that has greatly informed our understanding of the heliospheric interaction with the LISM, distinguishing particularly between solar minimum and maximum conditions. The first south polar observations occurred over the period June 26 to November 6, 1994, when the vehicle was above 70 degrees solar latitude. It reached a maximum of $80.2^{\circ}$ in September 1994. These observations were made during solar minimum and revealed very clearly the high speed solar wind and the low speed ecliptic wind. Ulysses passed the solar equator on March 5, 1995 on its way to traverse the north polar regions, which happened between June 19 and September 30, 1995, at a maximum latitude of $80.2^{\circ}$. As discussed above, Ulysses made important contributions to our understanding of the global heliospheric modulation of GCRs.

The Ulysses Mission was extended four times but eventually in early 2008, ESA and NASA terminated the mission with the communications systems failing and the RTGs deteriorating and thus power failing. Mission operations continued at reduced capacity until loss of contact June 30, 2009, more than 18.5 years after launch.

Ulysses carried a scientific payload of 10 instruments, some of which were of particular importance to our understanding of the heliospheric interaction with the LISM. These were the 1) Solar Wind Plasma Experiment (SWOOPS) (PI: D.J. McComas); 2) Magnetometer (MAG) (PI: A. Balogh); 3) Solar Wind Ion Composition Instrument (SWICS) (PI’s: J. Geiss, and G. Gloeckler); 4) Energetic Particle Instrument (EPAC) and GAS (PI: N. Krupp); 5) Low-Energy Ion and Electron Experiment (HI-SCALE) (PI: L.J. Lanzerotti); 6) Cosmic Ray and Solar Particle Instrument (COSPIN) (PI: R.B. McKibben), and 7) Dust Experiment (DUST) (PI: H. Krüger).

Note that the GAS instrument investigated neutral gas, especially helium, of interstellar origin. The SWICS instrument investigated the composition, temperature and speed of the atoms and ions that comprise the solar wind, of which the measurement of various pickup ion species was especially important in the heliosphere-LISM interaction context. DUST made direct measurements of interplanetary and interstellar dust grains, establishing their properties throughout the heliosphere.

**Interstellar Boundary Explorer – IBEX**: Interstellar Boundary Explorer, or IBEX, is a small explorer NASA mission led by Dr D.J. McComas (Mission PI) that brought an entirely new approach to investigating the interaction of the heliosphere with the LISM. IBEX carries two telescopes – instruments that gather energetic neutral atoms (ENAs) – named IBEX-Hi (PI: D.J. McComas) and IBEX-Lo (PI: S. Fuselier). The measured ENAs provide information about the heliospheric boundary, taking anything from 1 to 11 years to each 1 AU where they can be measured by IBEX-Lo or IBEX-Hi. The energy range measured by IBEX-Hi is 300 eV to 6 keV and for IBEX-Lo 10 eV to 2 keV.

Located at 1 AU, IBEX executes a 9-day orbit around the Earth. The launch of IBEX on 19 October 2008 was novel in that a Pegasus rocket carrying IBEX was dropped from a L–1011 aircraft to reach low–Earth orbit, after which a an additional solid rocket motor and its own onboard propulsion system to boosted IBEX into a high altitude orbit.

The IBEX mission has been scientifically highly productive and ground-breaking in its pioneering of a new observational technique for heliospheric science. A major discovery was the so-called IBEX Ribbon with the return of the first observations, a narrow region of intense ENA emission likely aligned with the interstellar magnetic field, a structure that had not been predicted in models and simulations of the heliospheric interaction with the LISM.

**Cassini**: The Cassini-Huygens mission, generally referred to as Cassini, was a collaborative mission between NASA, ESA, and the Italian Space Agency (ASI) to study Saturn and its system of rings and natural satellites. Launched on October 15, 1997, Cassini operated for nearly 20 years, 13 of which were spent orbiting Saturn and studying the planet and its system after entering orbit on July 1, 2004.

Of particular interest to heliospheric-LISM interaction science was the INCA sensor that was part of the Magnetospheric Imaging Instrument (MIMI; Krimigis et al. 2004) (PI: S.M. Krimigis) on the Cassini mission. INCA was a large geometry factor detector, capable of measuring the distributions of ENAs over the energy range of $< 7~\text{keV}$ to 3 MeV/nuc. and could analyze the composition (H and O groups), velocity, and direction of incident ENAs. Its purpose was to monitor the global dynamics of the Saturn-Titan magnetospheric system throughout the orbital tour. However, the INCA experiment exceeded the initial Cassini mission planning, being the first flown instrument to image the global heliosphere in 5.2–55 keV ENAs, providing insights on plasma processes at the distant boundaries of the heliosphere (Krimigis et al. 2009; Dialynas et al. 2017). The return of the first images from INCA over the period 2003 to 2009 revealed two unexpected heliospheric features (Dialynas et al. 2013): (a) The Belt, a broad band of ENA emission that wraps around the sky sphere, and (b) “Basins” where ENA minima occur. Notably, the INCA images date back to the year 2000 prior to Cassini’s encounter with Jupiter (Westlake et al. 2020).

**New Horizons**: The New Horizons mission, launched on January 19, 2006, is a NASA New Frontiers mission (PI: A. Stern) designed primarily to explore the Pluto system as well as other Kuiper Belt Objects. Despite its planetary focus, New Horizons carries four instrument suites of relevance to heliospheric-LISM science.

The Solar Wind Around Pluto (SWAP) instrument (PI: D.J. McComas) measures charged particles of up to 6.5 keV in energy. SWAP has returned the most important and detailed measurements of pickup ions in the distant heliosphere ever made. The Pluto Energetic Particle Spectrometer Science Investigation (PEPSSI) (PI: R. McNutt, Jr.) is a time of flight ion and electron sensor that measures charged particles up to 1 MeV energies. Alice (PI: A. Stern) is an ultraviolet imaging spectrometer that is now being used to investigate the Lyman-$\alpha $ backscatter glow and may have detected the H-wall. Finally, the Venetia Burney Student Dust Counter (VBSDC) (PI: M. Horanyi), built by students at the University of Colorado Boulder, operates periodically to make dust measurements including interstellar dust particles (in the range of nano- and picograms).

## The International Space Science Institute

The International Space Science Institute (ISSI) is an Institute of Advanced Study located in Bern, Switzerland. Scientists from around the world meet in a multi- and interdisciplinary setting to explore new scientific horizons. It is appropriate, given the auspices under which this summary of heliophysics-LISM research was conceived, to recall that the very first ISSI Workshop organized after the founding of ISSI in 1995 by the late Johannes Geiss was in fact the Workshop on the Interaction of the Heliosphere with the Local Interstellar Medium. The Workshop was organized by L.A. Fisk, J. Geiss, E. Grün, J. Lequeux, and E. Möbius and about 40 scientists attended the week-long November 1995 meeting. Indeed many of us on the author list, although much younger at the time, had the pleasure of attending the inaugural ISSI Workshop and the founding dinner. The Workshop set the tone for the publication policy and resulted in the first of many subsequent dedicated *Space Science Review* issues that were then collected into the now well-known volumes in the *ISSI Space Science Series* (von Steiger et al. 1996).

Subsequent meetings dedicated to the science of the heliosphere and its interaction with the LISM occurred in 2008 and 2010. All these meetings were of course in person. Sadly, this most recent of the ISSI Workshops dedicated to the heliosphere-LISM interaction some 25 years after the first Workshop and the founding of ISSI was disrupted by the COVID pandemic, necessitating the extensive use of virtual meetings for nearly two years. Nonetheless, fruitful and useful scientific exchanges were possible despite the circumstances being less than ideal and an excellent dedicated volume can be anticipated.
